# Cue-reactivity targeted smoking cessation intervention in individuals with tobacco use disorder: a scoping review

**DOI:** 10.3389/fpsyt.2023.1167283

**Published:** 2023-09-07

**Authors:** Miaoling Luo, Quan Gan, Yu Fu, Zhuangfei Chen

**Affiliations:** ^1^Medical School, Kunming University of Science and Technology, Kunming, China; ^2^Brain Science and Visual Cognition Research Center, Medical School of Kunming University of Science and Technology, Kunming, China; ^3^Faculté de Médecine, Université Paris-Saclay, Le Kremlin-Bicêtre, France

**Keywords:** tobacco use disorder, cue-reactivity, smoking cessation, intervention, cue-reactivity paradigms, scoping review

## Abstract

**Objectives:**

Cue-reactivity is a critical step leading to the emergence of addictive psychology and the triggering of addictive behaviors within the framework of addiction theory and is considered a significant risk factor for addiction-related behaviors. However, the effect of cue-reactivity targeted smoking cessation intervention and the cue-reactivity paradigms used in the randomized controlled trials varies, which introduces more heterogeneity and makes a side-by-side comparison of cessation responses difficult. Therefore, the scoping review aims to integrate existing research and identify evidence gaps.

**Methods:**

We searched databases in English (PubMed and Embase) and Chinese (CNKI and Wanfang) using terms synonymous with ‘cue’ and ‘tobacco use disorder (TUD)’ to April 2023, and via hand-searching and reference screening of included studies. Studies were included if they were randomized controlled trials taking cue-reactivity as an indicator for tobacco use disorder (TUD) defined by different kinds of criteria.

**Results:**

Data were extracted on each study’s country, population, methods, timeframes, outcomes, cue-reactivity paradigms, and so on. Of the 2,944 literature were retrieved, 201 studies met the criteria and were selected for full-text screening. Finally, 67 pieces of literature were selected for inclusion and data extraction. The results mainly revealed that non-invasive brain stimulation and exercise therapy showed a trend of greater possibility in reducing subjective craving compared to the remaining therapies, despite variations in the number of research studies conducted in each category. And cue-reactivity paradigms vary in materials and mainly fall into two main categories: behaviorally induced craving paradigm or visually induced craving paradigm.

**Conclusion:**

The current studies are still inadequate in terms of comparability due to their heterogeneity, cue-reactivity can be conducted in the future by constructing a standard library of smoking cue materials. Causal analysis is suggested in order to adequately screen for causes of addiction persistence, and further explore the specific objective cue-reactivity-related indicators of TUD.

## Introduction

“Substance addiction (or drug addiction) is a neuropsychiatric disorder characterized by a recurring desire to continue taking the drug despite harmful consequences.” (1), with tobacco being the most common and well-known addictive substance with a high risk of abuse (2). The World Health Organization’s Eighth Report on the Global Tobacco Epidemic (2021) pointed out that by 2019, the number of smokers over the age of 15 worldwide exceeded 1 billion, and the smoking rate reached 17.5%. Tobacco-induced diseases, such as lung cancer and diabetes, pose a significant threat to human health, causing 8 million yearly deaths worldwide (3).

Recent studies have shown that cue-induced cravings are crucial to address analyzing the physiological and neural processes that make it difficult to tobacco cessation implementation (4–6). And many studies found that cue-targeted interventions are effective in improving cessation outcomes (7–9). However, in regard to randomized controlled trials (RCTs) on tobacco use disorder (TUD), there are still unclearly and incompletely known (1) how many kinds of cue-reactivity targeted cessation interventions, (2) what effects these kinds of interventions have on cue-reactivity, and (3) what are the classification and content of smoking cue-reactivity paradigms. Therefore, an intimate understanding of the above issues will help to review the components of the smoking cessation intervention trials and provide insight into the reasons for trial heterogeneity.

### Cue-reactivity

Cue reactivity (CR) is a crucial characteristic of addiction (10). It is referred to “a phenomenon in which exposure to substance cues produces a range of physiological (e.g., alterations in heart rate, respiration, and temperature) and psychological (e.g., substance-related expectations and substance-relevant cognitive biases) responses, which motivates the individual to seek out and administer substances.” (11). In addition, CR is an essential factor in the onset of cravings (12) and may also be an effective predictor of relapse (13). Cue-reactivity in individuals with TUD is associated with tobacco relapse or persistent cessation (14–17). Individuals with TUD after abstinence could potentially relapse due to cravings triggered by re-exposure to smoking situations (18, 19). These situations are not limited to the actual smoking environment of tobacco, tobacco smells, tobacco images, and other scenes of smoking may also trigger a relapse (20, 21).

With advances in research methodology of quantitative cognitive science, an increasing number of researchers are exploring the relationship between measures of addictive behaviors (e.g., self-reported craving, efficacy assessments of tobacco cessation, prediction of relapse and so on) and neuroimaging biomarkers, for example, the activity of specific brain regions (e.g., insula and extended visual system) under cued responses may reflect addictive behaviors to some extent (22, 23) and may serve as the underlying neural basis for cued responses (24). Although functional magnetic resonance imaging (fMRI) brain responses are multiregional (23), and the electroencephalography (EEG) indicators (e.g., P300 and alpha power) from different types of smokers (e.g., early-onset and late-onset smokers) also vary (25–29), these findings can provide benchmarks as the theoretical tools for assessing smoking and formulating as well as improving personal tobacco cessation plans.

### CR indicators related to smoking cessation

Common indicators based on cue-reactivity assessment can be divided into three categories: psychological, physiological and neuroimaging indicators.

Psychological indicators could be subdivided into subjective and objective components, including subjective craving and impulsiveness, objective response inhibition, approach bias and attentional bias (30–38). Most studies have shown that smokers have increased subjective craving (30–32) and impulsiveness (33), as well as decreased inhibitory control (34, 35) and have selective approach bias (36) and attentional bias (37, 38) when exposed to smoking-related cues (SRC) compared to non-smokers.

Physiological indicators mainly include heart rate (HR), blood pressure (BP), sweat gland activity, skin temperature (ST), and skin conductance (SC). Carter et al. (12) found that HR [effect size (ES): *d* = 0.21] and sweat gland activity (ES: *d* = 0.44) increased in smokers compared to non-smokers in response to SRC, while ST (ES: *d* = 0.07) did not show statistically a significant difference between groups in most research among meta-analyzes. Betts et al. found (10) SC (ES: Hedges’ *g* = 0.19) had significant cue effects and non-significant physiological outcomes included HR, BP, electromyogram, salivation, ST, and startle reflex across studies. Therefore, the above suggests that these physiology-based studies have relatively small effects or no effects.

Studies of brain function primarily include fMRI and EEG indicators used to represent neural responses to SRC. A series of fMRI-based cue response studies found that smokers showed some activation or inhibition in various brain regions during SRC stimuli had been conducted and that there were correlations between specific brain networks, such as the mesolimbic system, medial prefrontal cortex (mPFC), insula, default mode network and salience network (22, 39–41). Engelmann et al. (23) found that smoking cues elicit larger fMRI responses than neutral cues in the extended visual system, precuneus, posterior cingulate gyrus, anterior cingulate gyrus, dorsal and mPFC, insula, and dorsal striatum. EEG-based cue-reactivity studies have shown that smokers exhibit specific changes in the EEG frequency band or event-related potential (ERP) component in response to SRC stimuli, such as the EEG power spectrum showing a significant increase in the alpha band or low-theta band coherence (25, 26). For ERP, the characteristic component is mainly P300. Compared to late smokers (age ≥ 16 years), early smokers (age < 16 years) have more robust P300 responses to smoking-related stimuli (27), and subjective craving is associated with a more substantial P300 amplitude, the higher impulsivity, the higher P300 amplitude (7, 27, 32). Another component is the late positive potential (LPP) of the ERP, which shows a greater LPP in response to smoking-related stimuli (28). The LPP induced by cigarette-related cues in a light smoke group that does not require a long smoking history can produce significant individual differences (29).

Since 1980, cue-reactivity-targeted indicators have been increasingly used to assess the effects of interventions for individuals with TUD (42, 43). The development of indicators based on cue-reactivity paradigms combined with pharmacology, neuroimaging is still an important focus in this field (4, 44). There are significant differences in the efficacy across tobacco cessation intervention studies. However, an overall comprehensive summary of the effects of various treatments and the cue-reactivity paradigms used in RCTs of cue-reactivity-targeted tobacco cessation interventions are still lacking. In this article, we present a scoping review, as being a precursor to systematic reviews, to explore more consistent findings and gaps in current research, to provide a rationale for the development of cue-reactivity-based valuation system for diagnosis and therapy.

## Methods

The scoping review was conducted according to Arksey and O’Malley’s framework: (1) identifying the research questions; (2) identifying studies; (3) selecting studies; (4) charting the data; and (5) collating, summarizing and reporting the results.

### Identifying research questions

This study specifies the questions for the scoping review: (1) what are the RCTs and the effects of cue-reactivity-targeted tobacco cessation interventions on TUD; (2) outline the cue-reactivity paradigms applied to tobacco cessation interventions on TUD.

### Search strategy

The search was conducted by combining subject terms and free words, using the Chinese search terms “smoking, cigarette addiction, tobacco addiction, cue” in the China National Knowledge Infrastructure (CNKI) and WanFang databases, the English search terms “tobacco, nicotine, cue” in the PubMed and EMBase databases. The main search of the database was performed in July 2021, the last update was in May 2022, and the final search of all databases was performed in April 2023. The specific search strategies for the four databases are described in Supplementary Table S1.

### Selection criteria

#### Inclusion criteria

1. The study population were individuals with TUD (comprehensively defined through every included literature which mentioned that their research subjects were cigarette or tobacco smokers who had certain score in FagerstrÖm Test for Nicotine Dependence (FTND) or met the criteria of whatever DSM-IV or − 5, or ICD-10, or not gave the detail diagnosis but just reported that the subjects were “nicotine dependence,” or “dependence smokers” or similar terms. See Supplementary Table S2 for detail);
2. The study design was an RCT;
3. The research topic was cue-reactivity as an indicator to evaluate the effects of smoking cessation.

#### Exclusion criteria

1. Literature other than English or Chinese;
2. Literature for which the full text cannot be obtained;
3. Literature with repeated publications;
4. Literature conducted only on animals or healthy subjects;
5. Literature recruiting subjects with multi-substance use disorder (e.g., cocaine, marijuana, heroin, methamphetamine, alcohol. See Supplementary Table S2 for detail) and/or related physical or mental illnesses (e.g., infectious diseases, cancer, schizophrenia. See Supplementary Table S2 for detail which also concludes the exclusion criteria of medication in each literature);
6. Literature that does not report outcomes in smokers exposed to tobacco-related cues;
7. Comment, research protocols, books or other non-scientific publications, case reports and conference abstracts.

After entering the retrieved literature titles into Endnote X9 for deduplication, a two-step review strategy was adopted: (1) title/abstract level; (2) full-text level. The two authors performed independent screening exercises. Disagreements between two authors (Luo and Gan) that emerged during the literature selection process would be discussed or consulted with a third author for consensus. Data for final inclusion in the literature were extracted and summarized in standardized tables. First author, time of publication, study sample and context, stimulus material, cue-reactivity paradigms, type of intervention, follow-up time, outcome measures and effects were extracted and recorded. The quality of evidence for each study and a formal risk of bias was not assessed. The data were aggregated and reported according to key themes.

In terms of outcome measures, we mainly focused on whether the difference between the treatment and control groups was statistically significant and whether the corresponding effect size was explicitly calculated in the included articles. When inter-group differences do not reach a significant level, we marked them as “NS” (not significant) and the corresponding effect size (ES) would not be shown in the tables, while when the inter-group differences reach a significant level, we would using the up or down arrow to show the change of the treatment compared to control group (s) and the corresponding ES would be shown in the tables. However, if the included articles do not report the ES, we would mark them as “NES” (no effect size). It is notable that a few articles only report the results of intra-group statistics, and in this case, we would provide descriptive comparison results of intra-group statistics.

### Patient and public involvement

Patients and/or the public were not involved in the design, conduct, reporting, or dissemination plans of this research.

## Results

### Study eligibility results

**Figure 1:**
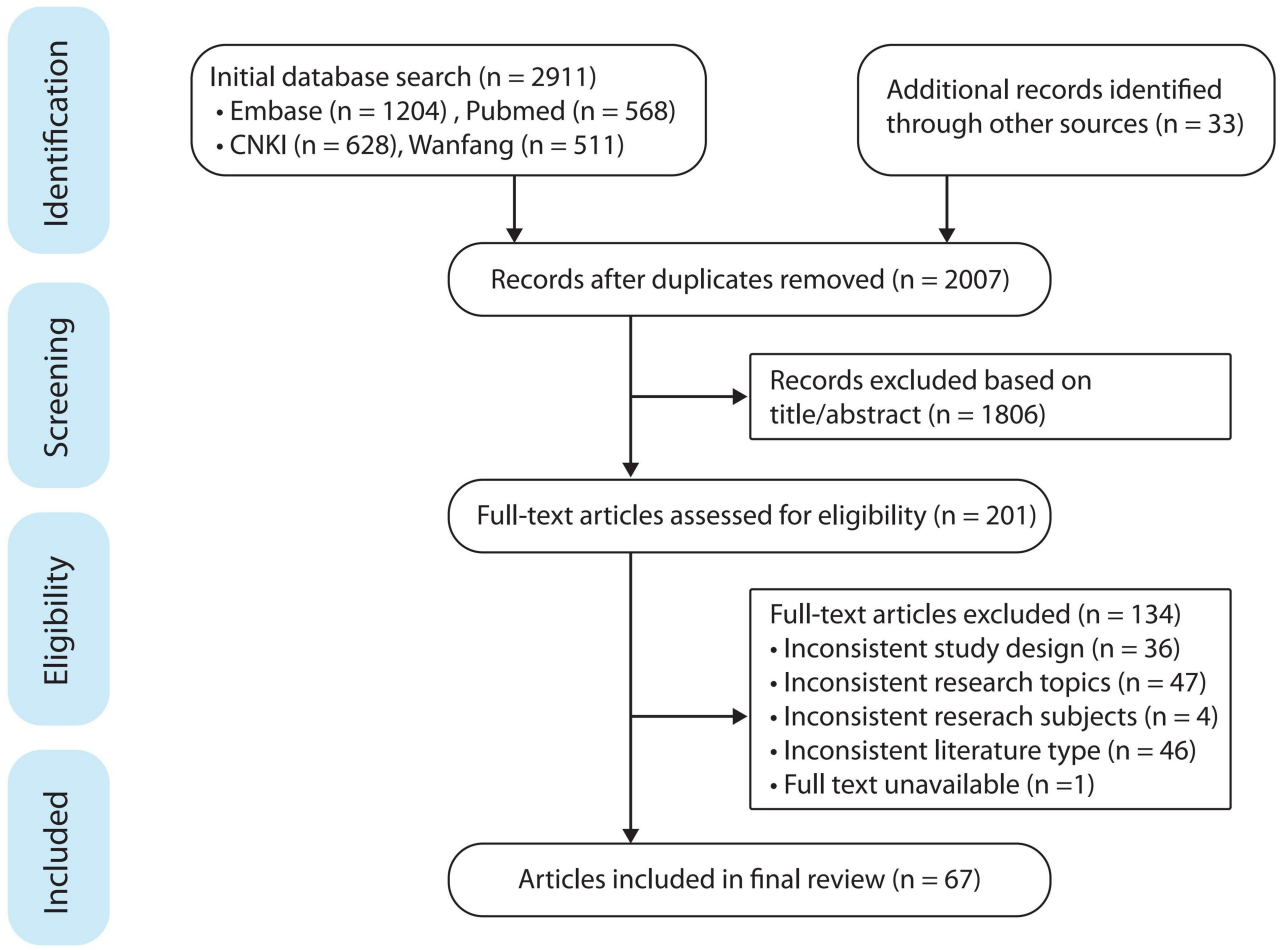
PRISMA flowchart.

Our research of PubMed and Embase databases in English, as well as CNKI and Wanfang search in Chinese, identified 2,911 possible records. After culling duplicates and checking abstracts and full-text records were confirmed. Finally, 67 records were included in the following analysis. The PRISMA flowchart is given in Figure 1.

### Basic information on included studies

The results of the current scoping review identified 67 RCTs covering tobacco cessation therapy, 28 articles of pharmacotherapy, 9 articles of physiotherapy, 11 articles of psychotherapy, 6 articles of exercise therapy and 13 articles of other therapies (primarily combination therapy), respectively. More than half of the included studies were conducted in the United States (one of them is from a multicentre study; *n* = 42) (8, 9, 45–84), while a minority were conducted in the United Kingdom (*n* = 6) (85–90), Canada (*n* = 6) (91–96), China (n = 4) (7, 97–99), Brazil (*n* = 2) (100, 101), Israel (one of them is from a multicentre study mentioned above; *n* = 2) (9, 102), Netherlands (*n* = 2) (103, 104), Chile (*n* = 1) (105), Korea South (*n* = 1) (106), Germany (*n* = 1) (107), and France (*n* = 1) (108). Basic information from the included literature is shown in Tables 1–5.

**Table 1:** Details of 28 included studies that looked at pharmacotherapy that modulates cue reactivity.

Authors (year)	Sample and context	Sex and age/year /M (SD)	Control group (s)	Intervention method and follow-up time	Smoking-CR related outcome measure and intervention effect		
					Subjective or behavioral measures	Physiological responses	Brain imaging
Tiffany et al. (2000) (70)	61 cigarette smokers who were not attempting to quit smoking; United States	31 females; ≥21, 31.5 (NG)	A placebo patch	Transdermal nicotine patch; (No)	(NES) (NS): craving (QSU-Brief); affect (Mood Form)	(NES) (NS): skin conductance; heart rate	NA
Shiffman et al. (2003) (71)	296 smokers who were motivated to quit smoking; United States	173 females; 18–65, 39.3 (11.3)	Control gum	Nicorette gum (4 mg for heavy smokers; 2 mg for light smokers; in the original flavor); (No)	(NES): craving (5-item 100-mm VAS)↓	NA	NA
Hutchison et al. (2004) (57)	59 cigarette smokers; United States	25 females; NG, placebo (*n* = 28): 20.6 (3.9), olanzapine (*n* = 31): 22.3 (4.1)	Placebo group	Olanzapine (5 mg/d, 5 d); (No)	(NES): craving (5-item 100-mm VAS)↓; affect (PANAS) (NS)	NA	NA
Waters et al. (2004) (72)	158 smokers who had to report high motivation and efficacy to quit; United States	82 females; NG, 38.6 (9.5)	Placebo group	Nicotine patches (35 mg); (No)	(ES) (NS): urge (a 10-point scale ranging from 0 to 9); reaction time response (CR task)	NA	NA
Mahler et al. (2005) (58)	20 cigarette smokers; United States	6 females; NG, 27.4 (5.0)	Placebo group	Haloperidol (2 and 4 mg); (No)	(NES) (NS): craving (QSU-Brief)	(NES): heart rate: 2 mm haloperidol↓, 4 mg haloperidol (NS)	NA
Morissette et al. (2005) (73)	52 smokers not currently considering quitting or changing their smoking; United States	29 females; ≥18, 20.6 (1.7)	Placebo patch group	Nicotine patch (21 mg); (No)	(NES) (NS): craving (QSU-Brief)	NA	NA
Niaura et al. (2005) (74)	319 smokers not trying to quit smoking; United States	188 females; 18–65, 37.79 (13.13)	Nicotine polacrilex gum	Rapid-release nicotine gum; (No)	(NES): craving (5-item 100-mm VAS)↓	NA	NA
Reid et al. (2007) (59)	40 smokers interested in quitting smoking; United States	15 females; NG, topiramate (*n* = 19): 43.0 (13.7), placebo (*n* = 21)	Placebo group	Topiramate (75 mg/day, 9 d); (No)	(NES) (NS): smoking urge (QSU-Brief); withdrawal (WST)	(NES) (NS): skin conductance; skin temperature; heart rate; blood pressure	NA
Liu et al. (2009) (97)	20 male smokers; China	Only males; 21–45, 10 light smokers: 29.40 (2.07), 10 heavy smokers: 30.10 (1.64)	Each participant served as their own control	Aripiprazole (placebo, 5, and 10 mg); (No)	(NES): smoking urge (QSU-Brief: heavy smokers with placebo↑, light smokers with 10 mg aripiprazole↑); craving (VAS) (NS); smoking withdrawal symptoms (WST) (NS)	(NES): heart rate (NS), blood pressure (administration of 10 mg aripiprazole: diastolic pressure↓)	NA
Hussain et al. (2010) (91)	24 current daily smokers not trying to quit or reduce smoking; Canada	13 females; NG, 40.1 (9.8)	Placebo group	Bupropion SR (300 mg/day, 6 weeks); (2 weeks)	(ES) (NS): craving (VAS, TCQ)	NA	NA
Brandon et al. (2011) (60)	100 smokers; United States	39 females; 18–60, varenicline (*n* = 46): 45.8 (9.4), placebo (*n* = 54): 41.2 (11.5)	Placebo group	Varenicline; (No)	(NES): craving (a single item using 0 to 20 scale: how strong was your craving to smoke a cigarette?)↓ only at assessment 3	(NES): heart rate↓ only at assessment 3; skin conductance (NS)	NA
Culbertson et al. (2011) (61)	30 treatment-seeking cigarette smokers; United States	9 females; NG, bupropion (*n* = 14): 40.4 (2.8), placebo (*n* = 16): 42.9 (3.1)	Placebo group	Bupropion SR; (No)	(NES): craving (1 to 5 on the question, “I crave a cigarette right now”)↓ during the crave-resist condition	NA	fMRI (NES): brain activity in left *VS*↓, right mOFC↓, and bilateral ACC↓
Franklin et al. (2011) (45)	16 smokers not currently considering quitting; United States	Only males; NG, 36.1 (2.2)	Placebo group	Varenicline; (No)	(NES) (NS): craving (SJWS)	NA	fMRI (NES): brain activity in *VS*↓ and mOFC↓
Ditre et al. (2012) (62)	72 treatment-seeking smokers; United States	43 females; 18–65, 43.46 (10.96)	Placebo group	Divalproex; (18 weeks)	(NES): craving (VAS)↑; subjective emotional responses (arousal, SAM) (NS)	(NES) (NS): heart rate, skin conductance, facial electromyography, startle response	NA
Hitsman et al. (2013) (46)	38 non-treatment-motivated smokers; United States	NG; 18–65, 36.2 (14.4)	Placebo group	Varenicline; (1 week)	(NES) (NS): craving (5-item scale), withdrawal (MNWS), affect (PANAS)	NA	NA
Du et al. (2014) (77)	322 low dependence smokers; United States	163 females; ≥18, 2.5 mg nicotine film (*n* = 161): 39.0 (12.9), 2 mg nicotine lozenge (*n* = 160): 39.8 (12.6)	2 mg nicotine lozenge	Nicotine oral soluble film (2.5 mg); (No)	(NES): craving (0-100 mm, VAS)↓	NA	NA
Rabinovitz et al. (2014) (102)	48 regular cigarette smokers not interested in quitting; Israel	32 females; 18–45, 29.1 (6.7)	Placebo group	Omega-3 fatty acids; (30 days)	(NES): craving (TCQ-SF)↓	NA	NA
Schlagintweit et al. (2014) (94)	70 non-treatment-seeking smokers; Canada	34 females; 19–57, 27 (9.2)	Placebo group	Nicotine lozenge (4 mg); (No)	(NES) (NS): craving (QSU-Brief)	(NES) (NS): heart rate	NA
Pachas et al. (2015) (47)	74 smokers; United States	20 females; 18–65, propranolol (*n* = 35): 41.6 (10.9), placebo (*n* = 39): 42.5 (9.8)	Placebo group	Propranolol; (1 week)	(NES) (NS): emotional state (using a 13-point Likert-type rating scale), craving (using an 8-point Likert-type rating scale)	(NES) (NS): skin conductance, heart rate, left corrugator electromyogram	NA
Haarmann et al. (2016) (107)	17 smokers; Germany	9 females; 25–60, placebo (*n* = 9): 42.4 (3.6), varenicline (*n* = 8): 44.5 (3.8)	Placebo group	Varenicline; (6 weeks)	NA	(NES): muscle sympathetic nerve activity (NS), baroreflex sensitivity (NS), heart rate↓, blood pressure (NS)	NA
Miller et al. (2016) (63)	17 daily cigarette smokers; United States	6 females; 18–35, 23.5 (4.2)	Placebo nasal sprays (each participant served as their own control)	Intranasal oxytocin (40 IU); (No)	(ES): craving (TCQ-SF, QSU-Brief)↓ (η^2^_*p*_ = 0.2)	NA	NA
Gendy et al. (2018) (92)	27 treatment-seeking smokers; Canada	10 females; 19–65, 43 (12)	Placebo group (each participant served as their own control)	Gemfibrozil; (No)	(NES) (NS): craving (TCQ-SF, VAS), affect (MF)	(NES) (NS): heart rate, skin temperature, blood pressure, skin conductance	NA
Nides et al. (2018) (56)	Study 1: 187 smokers; United States	84 females; ≥18, heavy smokers: 4-mg nicotine mini lozenge (*n* = 52): 45.1 (10.4), placebo (n = 34): 45.9 (9.4); light smokers: 1.5-mg nicotine mini lozenge (*n* = 60): 43.9 (11.5), placebo (*n* = 41): 45.0 (10.6)	Matching placebo group	1.5-mg or 4-mg nicotine mini lozenge; (No)	(NES) (NS): craving (a 100-mm VAS, using a questionnaire consisting of the following 5 items)	NA	NA
	Study 2: 323 smokers; United States	149 females; ≥18, 4-mg nicotine mini lozenge (*n* = 162): 43.6 (12.1), placebo (*n* = 161): 44.5 (11.6)	Matching placebo group	4-mg nicotine mini lozenge; (No)	(NES): 5 min post-treatment craving (using the same questionnaire as Study 1) (least-square mean [LSM])↓	NA	NA
Versace et al. (2019) (48)	210 smokers attempting to quit; United States	74 females; 18–65, 44.6 (10.7)	Placebo group	Varenicline tartrate; Bupropion SR; (4 weeks)	NA	NA	EEG (NES) (NS): LPP amplitude
Ketcherside et al. (2020) (8)	43 non-abstinent, sated treatment-seeking cigarette smokers; United States	16 females; 18–60, baclofen (*n* = 21): 38.9 (12), placebo (*n* = 22): 40.2 (12)	Placebo group	Baclofen; (No)	(NES): craving (CWQ)↓	NA	fMRI (NES): resting brain activation of the right DLPFC↑; neural response in the vmPFC↓ and left avInsula↓
Kotlyar et al. (2020) (80)	58 smokers; United States	29 females; 18–64, 42.6 (13.1)	Placebo or no treatment prior to cue exposure	4 mg nicotine lozenge 15 min prior to cue exposure; (No)	Versus no treatment group (ES): craving (MNWS: ES = 0.60, QSU-Brief: ES = 0.7)↓, withdrawal (MNWS: ES = 0.37)↓	NA	NA
Lawson et al. (2021) (49)	161 treatment-seeking smokers; United States	95 females; 18–70, varenicline group (*n* = 82): 55.2 (9.5), placebo group (*n* = 79): 55.0 (9.2)	Placebo group	Varenicline; (No)	(NES) (NS): craving (using a 4-item craving scale derived from the QSU)	NA	NA
Novick et al. (2022) (82)	39 smokers not currently seeking treatment for nicotine dependence; United States	21 females; 18–50, 36.1 (8.7)	Placebo group	Progesterone; (No)	NA	NA	fMRI (NES) (NS between males and females): neural activation (ACC, PCC, LLOC, LMTG)

M, mean; SD, standard deviation; CR, cue reactivity; NA, no assessment; NS, no significant; NG, not given; ES, effect size; NES, no effect size; QSU-Brief, the 10-item (brief version of) Questionnaire of Smoking Urges; VAS, Visual Analog Scale; SJWS, Shiffman-Jarvik Withdrawal Scale; fMRI, functional Magnetic Resonance Imaging; WST, withdrawal scale of tobacco; ES, effect size; TCQ, Tobacco Craving Questionnaire; SR, sustained release; VS, ventral striatum; mOFC, medial orbitofrontal cortex; ACC, anterior cingulate cortex; SAM, Self Assessment Manikin; MNWS, Minnesota Nicotine Withdrawal Scale; PANAS, Positive and Negative Affect Scale; TCQ-SF, Tobacco Craving Questionnaire-Short Form; MF, Mood Form; EEG, electroencephalography; LPP, late positive potential; CWQ, Craving and Withdrawal Questionnaire; DLPFC, dorsolateral prefrontal cortex; vmPFC, ventromedial prefrontal cortex; avInsula, anterior ventral insula; PCC, posterior cingulate cortex; LLOC, left lateral occipital cortex; LMTG, left middle temporal gyrus. The symbols “↑, ↓” indicate increasing (↑) and decreasing (↓) respectively.

**Table 2:** Details of 9 included studies that looked at noninvasive brain stimulation that modulates cue reactivity.

Authors (year)	Sample and context	Sex and age/year /M (SD)	Control group (s)	Intervention method and follow-up time	Smoking CR-related outcome measure and intervention effect		
					Subjective or behavioral measures	Physiological responses	Brain imaging
Fregni et al. (2008) (100)	24 smokers; Brazil	11 females; 18–55, 24.8 (7.6)	Sham tDCS	Anodal tDCS of the left and right DLPFC; (No)	(NES): craving (VAS, 5 items)↓	NA	NA
Boggio et al. (2009) (50)	27 regular smokers; United States	12 females; 18–55, 26.3 (8.4)	Sham tDCS	Anodal tDCS of the left DLPFC for 5 consecutive days: a constant current of 2 mA intensity was applied for 20 min; (No)	(NES): craving (VAS, 5 items)↓	NA	NA
Li et al. (2013) (64)	16 non-treatment-seeking smokers; United States	4 females; 21–60, 42.6 (11.5)	Active sham TMS	One session of high-frequency rTMS (10 Hz, 100% resting motor threshold, 5 s-on, 10 s-off, 60 trains, 3,000 pulses, 15 min) over the left DLPFC; (No)	(NES): craving (QSU-Brief)↓	NA	NA
Meng et al. (2014) (98)	30 smokers; China	Only male; 18–55, 23.7 (7.2)	Sham tDCS	Cathodal tDCS (bilateral cathodal stimulation of the FPT area or cathodal over right FPT); (No)	(NES) (NS): attentional bias	NA	NA
Li et al. (2017) (109)	11 non-treatment seeking nicotine dependent cigarette smokers; United States	6 females; 18–60, 39.7 (13.2)	Sham rTMS (each participant served as their own control)	One session of active rTMS (10 Hz, 5 s-on, 10 s-off, 100% motor threshold, 3,000 pulses) over the left DLPFC; (No)	(NES) (NS): craving (0–10, VAS)	NA	fMRI (NES): BOLD activation in contralateral mOFC↓ and ipsilateral NAc↓
Yang et al. (2017) (99)	32 chronic smokers; China	Only male; NG, 26.68 (6.28)	Sham tDCS (each participant served as their own control)	tDCS over left DLPFC; (No)	(ES): craving (VAS)↓ (d = 0.410)	NA	fMRI(ES): ↑modulating the coupling between DLPFC and PHG (*d* = 0.589)
Li et al. (2020) (65)	42 treatment-seeking nicotine-dependent smokers; United States	21 females; 18–60, sham rTMS (*n* = 17): 44.12 (9.1), active rTMS (*n* = 21): 41.19 (11.8)	Sham rTMS	Ten daily sessions of rTMS over the left DLPFC; (3 months)	(NES): craving (QSU-Brief/VAS)↓, withdrawal symptoms (MNWS) (NS)	NA	NA
Zangen et al. (2021) (9)	262 chronic smokers who had made at least one prior failed attempt to quit, with 68% having made at least three failed attempts; the US (12 sites) and Israel (two sites)	126 females; 22–70, active (*n* = 123): 45.0 (13.0), sham (*n* = 139): 44.8 (13.4)	Sham rTMS	60 rTMS trains of 30 pulses were applied at 10 Hz (3 s each train) with 15 s intertrain intervals, bilaterally stimulate neuronal pathways in the lateral prefrontal cortex and insula with an intensity above the neuronal threshold for activation; (15 weeks)	(NES): craving (VAS)↓	NA	NA
Marques et al. (2022) (101)	24 smokers; Brazil	9 females; 18–70, NG	1 Hz rTMS of primary motor cortex group	A single session of 1 Hz rTMS over left frontal pole; (No)	(NES) (NS): craving (items 1, 3, 5, 6 and 7 of QSU-Brief) shown together with a 1–7 analog visual scale	NA	NA

M, mean; SD, standard deviation; CR, cue reactivity; NA, no assessment; NS, no significant; NG, not given; ES, effect size; NES, no effect size; tDCS, transcranial direct current stimulation; DLPFC, dorsolateral prefrontal cortex; VAS, Visual Analog Scale; rTMS, repetitive transcranial magnetic stimulation; fMRI, functional Magnetic Resonance Imaging; BOLD, blood oxygen level dependent; mOFC, medial orbitofrontal cortex; NAc, nucleus accumbens; QSU-Brief, the 10-item (brief version of) Questionnaire of Smoking Urges; FPT, frontal–parietal–temporal; PHG, para-hippocampal gyrus; MNWS, Minnesota Nicotine Withdrawal Scale. The symbols “↑, ↓” indicate increasing (↑) and decreasing (↓) respectively.

**Table 3:** Details of 11 included studies that looked at psychotherapy that modulates cue reactivity.

Authors (year)	Sample and context	Sex and age/year /M (SD)	Control group (s)	Intervention method and follow-up time	Smoking CR-related outcome measure and intervention effect		
					Subjective or behavioral measures	Physiological responses	Brain imaging
Bowen et al. (2009) (52)	123 smokers who were interested in changing their smoking; United States	33 females; ≥18, 20.33 (3.34)	A no-instruction control group	Brief mindfulness-based intervention; (7 days)	(NES) (NS): urge (QSU-Brief)	NA	NA
Kim et al. (2015) (106)	14 smokers who were motivated to quit smoking but were not currently undergoing any treatment; Korea	Only male; NG, activity-based NF (*n* = 7): 26.00 (2.16), FC-added NF (*n* = 7): 26.00 (1.29)	Activity-based rtfMRI-NF	FC-added rtfMRI-neurofeedback; (No)	(NES) (NS): craving (current craving score 1–10 by pressing a button on a fiber-optic response pad)	NA	fMRI (NES): neuronal activity↑ and functional connectivity↑ between the targeted ROIs (ROIs1: ACC and medial pFC, ROIs2: posterior cingulate cortex and precuneus)
Elfeddali et al. (2016) (103)	434 smokers not having made a quit-attempt yet; Netherlands	299 females; 18–65, 40.76 (11.04)	Placebo-training	A multiple-sessions Web-based ABM self-help intervention; (6 months)	(NES) (NS): cognitive biases (attentional bias, approach bias)	NA	NA
Hartwell et al. (2016) (66)	44 smokers not seeking treatment; United States	16 females; 18–60, feedback group (*n* = 21): 34.1 (11.3), control group (*n* = 23): 36.2 (10.6)	A no-feedback control group	rtfMRI-neurofeedback; (1 week)	(NES): urge (QSU-Brief)↓	NA	fMRI (NES): craving-related ROI (PFC) activation↓
Froeliger et al. (2017) (53)	13 smokers; United States	4 females; ≥18, 49 (12.2)	A demographically matched comparison group	8-weeks of mindfulness-oriented recovery enhancement; (No)	(ES) (NS): craving (modified version of SJWQ)	NA	fMRI (ES): CR-BOLD↓ response in *VS* (*d* = 1.57) and vPFC (*d* = 1.7); rsFC↑ between rACC and OFC
Germeroth et al. (2017) (67)	88 treatment-seeking cigarette smokers; United States	31 females; 18–65, R-E group (*n* = 44): 48.3 (12.5), NR-E group (*n* = 43): 46.7 (12.8)	Nonsmoking-related retrieval followed by extinction training	A brief memory updating intervention (retrieval-extinction training); (1 month)	(ES) (NS): craving (self-report craving questionnaire); negative affect (a modified version of the self-report Mood Form)	(ES) (NS): heart rate, blood pressure	NA
Andreu et al. (2018) (105)	50 smokers who were interested in cutting down or quitting smoking; Chile	33 females; ≥18, mindfulness group (*n* = 25): 20.0 (1.72), control group (*n* = 25): 20.6 (1.75)	A group receiving control-instructions (for 15 min approximately)	A brief mindfulness-meditation intervention; (No)	(NES) (NS): craving (QSU-Brief); error rates and reaction times on the smoking Go/NoGo	NA	EEG (NES): P3 amplitude↓ and N2 amplitude (NS) during NoGo vs. Go trials
Bu et al. (2019) (7)	60 smokers; China	Only males; 18–40, real-feedback group (*n* = 28): 23.7 (3.8), yoked-feedback group (*n* = 25): 23.4 (3.1)	Neurofeedback training from yoked-feedback	Neurofeedback training from real-feedback; (4 months)	(ES): craving (TCQ)↓ (d = 0.61)	NA	EEG (ES): P300 amplitude↓ (ES: *d* = 0.64)
Malbos et al. (2022) (108)	100 smokers; France	71 females; ≥18, 47.65 (13.31)	Cognitive behavioral therapy (CBT)	Virtual reality cue exposure therapy (VRCE); (No)	(NES) (NS): craving (French Tobacco Craving Questionnaire; VAS)	NA	NA
Yang et al. (2022) (83)	129 daily smokers motivating to quit smoking; US	60 females; ≥18, 47.6 (13.4)	Control condition	A single cue-exposure session with augmented reality (AR) cigarette cues (extinction condition); (No)	(ES) (NS): urge (VAS)	NA	NA
Barnabe et al. (2023) (96)	62 non-treatment seeking smokers; Canada	28 females; 18–65, 35.82 (12.99)	Non-stressful condition	Stress-based intervention (stress and smoking cues were combined in a memory updating); (6 weeks)	(NES) (NS): craving (TCQ-SF and QSU-Brief)	(NES) (NS): blood pressure; skin conductance; heart rate	NA

M, mean; SD, standard deviation; CR, cue reactivity; NA, no assessment; NS, no significant; NG, not given; ES, effect size; NES, no effect size; QSU-Brief, the 10-item (brief version of) Questionnaire of Smoking Urges; ROI, region of interest; FC-added rtfMRI-NF, real-time fMRI neurofeedback approach that includes a functional connectivity component; fMRI, functional Magnetic Resonance Imaging; ACC, anterior cingulated cortex; ABM, Attentional Bias Modification; SJWQ, Shiffman-Jarvik Questionnaire; BOLD, blood oxygen level dependent; VS, ventral striatum; vPFC, ventral prefrontal cortex; rsFC, resting-state functional connectivity; rACC, right rostral anterior cingulate cortex; OFC, orbital frontal cortex; EEG, electroencephalography; the R-E group, smoking-related memory retrieval followed by extinction training; the NR-E group, nonsmoking-related retrieval followed by extinction training; TCQ, Tobacco Craving Questionnaire; TCQ-SF, Tobacco Craving Questionnaire-Short Form. The symbols “↑, ↓” indicate increasing (↑) and decreasing (↓) respectively.

**Table 4:** Details of 6 included studies that looked at exercise therapy that modulates cue reactivity.

Authors (year)	Sample and context	Sex and age/year/ M (SD)	Control group (s)	Intervention method and follow-up time	Smoking CR-related outcome measure and intervention effect		
					Subjective or behavioral measures	Physiological responses	Brain imaging
Taylor et al. (2007) (85)	60 smokers; United Kingdom	34 females; NG, exercise condition (*n* = 31): 27.1 (5.5), passive condition (*n* = 29): 30.1 (9.7)	Passive condition (seating quietly)	A 15-min brisk walk; (No)	(ES): craving (single item “I have a desire for a cigarette right now” using a 7-point scale)↓ (1 < d < 2); strength of desire to smoke (a 7-point scale)↓ (*d* = 0.42); smoking withdrawal symptoms (MPSS) of depression (NS), tension↓ (*d* = 0.69), irritability (NS), restlessness (NS), poor concentration↓ (*d* = 0.44), stress↓ (*d* = 0.67), and anxiety (NS)	NA	NA
Janse Van Rensburg et al. (2009a) (86)	10 smokers who were not currently making an attempt at smoking cessation; United Kingdom	4 females; 18–50, NG	Passive control condition (passive seating for 10 min) (each participant served as their own control)	An exercise (10 min moderate-intensity stationary cycling); (No)	(ES): cravings (a seven-point scale for the item “I have a desire to smoke”)↓ (η^2^ = 0.573)	NA	fMRI (NES):, brain default mode (Broadmanns Area 10)↑
Janse Van Rensburg et al. (2009b) (87)	20 smokers not currently making an attempt to quit smoking; United Kingdom	5 females; 18–50, 29.05 (9.37)	Passive control condition (passive seating for 15 min)	15 min of exercise (moderate intensity stationary cycling); (No)	(NES): craving (a seven-point scale for the single item “I have a desire to smoke right now”)↓; attentional biases (% dwell time on smoking images↓, direction of initial fixations to smoking images↓)	NA	NA
Elibero et al. (2011) (68)	76 daily smokers not currently engaged in an attempt to quit smoking; United States	28 females; 18–45, cardiovascular exercise (*n* = 25): 28.36 (7.4), Hatha yoga (*n* = 26): 30 (9.2), no-exercise (*n* = 25): 28.28 (8.7)	A nonactivity control condition group	A 30-min bout of cardiovascular exercise (brisk walk on a treadmill) or Hatha yoga; (No)	(NES): craving (0–20 scale)↓ only at cardiovascular exercise group	NA	NA
Janse Van Rensburg et al. (2013) (54)	162 smokers who were not making a current quit attempt; United States	55 females; 18–50, 30.8 (9.8)	Passive control (watching an educational video about the health benefits of exercise)	Light and vigorous intensity aerobic exercise; (No)	(NES) (NS): craving (QSU-brief)	(NES): startle reflex magnitude↓ in vigorous exercise	NA
Fong et al. (2014) (93)	25 smokers; Canada	14 females; 18–65, experimental (*n* = 12): 35.7 (14.9), control (*n* = 13): 39.1 (15.2)	Passive control (seating alone in a quiet room for 15 min)	A single, 15-min bout of moderate intensity exercise; (No)	(NES): craving (a single item “How strong is your desire to smoke right now?” and is scored on a 7-point Likert scale)↓, psychological withdrawal symptoms (SJWS)↓	NA	NA

M, mean; SD, standard deviation; CR, cue reactivity; NA, no assessment; NS, no significant; NG, not given; ES, effect size; NES, no effect size; MPSS, Mood and Physical Symptoms Scale; fMRI, functional Magnetic Resonance Imaging; QSU-Brief, the 10-item (brief version of) Questionnaire of Smoking Urges; SJWS, Shiffman-Jarvik Withdrawal Scale. The symbols “↑, ↓” indicate increasing (↑) and decreasing (↓) respectively.

**Table 5:** Details of 13 included studies that looked at other therapies that modulate cue reactivity.

Authors (year)	Sample and context	Sex and age/year /M (SD)	Control group (s)	Intervention method and follow-up time	Smoking CR-related outcome measure and intervention effect		
					Subjective or behavioral measures	Physiological responses	Brain imaging
Hutchison et al. (1999) (69)	20 regular smokers with some motivation to quit; United States	10 females; NG, placebo+TNR: 37.2 (10.9), naltrexone+TNR: 42.2 (9.7)	Placebo group	Naltrexone+TNR; (No)	(ES) (NS): urge to smoke (a single item scale from 0 to 100); affect (PANAS); withdrawal (an updated version of the Minnesota Withdrawal scale)	NA	NA
Sayette et al. (1999) (55)	58 smokers; United States	28 females; 18–35, 22.7 (3.2)	Control odor (sniffed distilled water)	Olfactory stimuli: a pleasant or unpleasant odor; (No)	(NES): urge (a scale of 0–100, “0 = no urge at all, 100 = the strongest urge I’ve ever felt”)↓	NA	NA
Rohsenow et al. (2007) (75)	134 smokers not currently trying to quit smoking; United States	62 females; NG, men (*n* = 72): 49.3 (12.4), women (*n* = 62): 44.5 (11.7)	Double-placebo group	TNR (42/21/0 mg patch) with naltrexone (50 mg) or placebo; (No)	(ES): urge (QSU-Brief)↓ (42 mg TNR); nicotine withdrawal (MNWS)↓ (42 mg TNR + naltrexone, *f* = 0.56)	(ES): (42/21 mg TNR): heart rate↑; mean arterial pressure↑	NA
Santa Ana et al. (2009) (76)	25 smokers; United States	13 females; 18–55, 41.3 (9.1)	Placebo plus cue exposure therapy group	DCS combined with cue exposure treatment; (1-and 4-week)	(NES): urge (10-point Likert scale and QSU-Brief)↓	(NES): skin conductance↓ (only at experimental sessions 2)	NA
Kamboj et al. (2012) (88)	32 non-treatment-seeking heavy smokers; United Kingdom	10 females; 18–65, placebo (*n* = 16): 32.31 (10.64), DCS (*n* = 16): 30.00 (9.37)	Placebo group	2 sessions of exposure/response prevention combined with DCS; (2 weeks)	(NES) (NS): craving (0-100 mm, VAS); attentional bias	(NES) (NS): skin conductance	NA
Havermans et al. (2014) (104)	48 male smokers; Netherlands	Only males; NG, NicVAX (*n* = 20): 33.4 (7.1), placebo (*n* = 13): 28.8 (7.1)	Placebo group	Five injections with 400 μg/mL of the NicVAX; (No)	NA	NA	fMRI (NES) (NS): brain activity to smoking cues
Begh et al. (2015) (90)	118 smokers attempting cessation; United Kingdom	69 females; ≥18, 44.8 (12.7)	Placebo training++21 mg nicotine patches (from quit day onwards) and behavioral support	Five weekly sessions of attentional retraining+21 mg nicotine patches (from quit day onwards) and behavioral support; (6 months)	(NES) (NS): craving (MPSS); attentional bias	NA	NA
Das et al. (2015) (89)	59 motivated to quit smokers; United Kingdom	NG; 18–65, MEM + REACT (*n* = 19): 29.32 (9.9), PLAC+REACT (*n* = 20): 28.35 (7.04), MEM no REACT (*n* = 20): 27.45 (6.91)	PLAC+REACT; MEM no REACT	Memantine in combination with smoking memory reactivation; (3 months)	(NES) (NS): craving (single-item 100-mm VAS); attentional bias	(NES) (NS): blood pressure; skin conductance; heart rate variability	NA
Schlagintweit et al. (2016) (95)	30 dependent smokers; Canada	13 females; 20–58, 26.37 (7.28)	Denicotinized cigarette group	Acute tobacco smoking (nicotine-containing cigarette); (No)	(NES) (NS): craving (QSU-Brief)	(NES) (NS): heart rate	NA
Jones et al. (2017) (78)	27 cigarette smokers not interested in smoking cessation; United States	2 females; 21–55, pioglitazone 0 mg (*n* = 13): 41.6 (10.0), pioglitazone 45 mg (*n* = 14): 44.9 (6.7)	Placebo+a transdermal nicotine patch group	Pioglitazone+a transdermal nicotine patch; (No)	(ES) (NS): craving (QSU)	(ES): skin conductance↑; skin temperature (NS); heart rate (NS)	NA
Brandon et al. (2018) (79)	58 cigarette smokers; United States	25 females; ≥18, 50.5 (10.8)	Standard Varenicline or extended Varenicline	Extended Varenicline+facilitated extinction; (3 months)	(NES) (NS): craving (VAS)	NA	NA
Otto et al. (2019) (81)	62 smokers who expressed a desire to quit smoking; United States	44 females; 18–65, (NG)	Cognitive-behavioral treatment combined with nicotine replacement therapy+placebo prior to each of two sessions of CET	CBT and varenicline+DCS prior to each of two sessions of CET; (6 weeks)	(ES): craving (VAS)↓ (*d* = 1.21)	(ES): skin conductance↓ (ES: 1.45), heart rate (NS), left corrugator electromyogram (NS)	NA
Robinson et al. (2022) (84)	246 treatment-seeking smokers; United States	89 females;18–65, 46.28 (10.93)	8 weeks of NRT after completing sham training using the “unmodified” dot-probe task (i.e., both cue types probed equally to avoid ABM)	8 weeks of NRT after completing smartphone-delivered, in-home ABM administered using the modified dot-probe task (i.e., neutral cues probed 100% of the time to train attentional bias away from smoking cues); (8 weeks)	(ES): attentional bias↓ (η^2^_*p*_= 0.08 at 1-day posttraining and η^2^_*p*_ = 0.04 at 8 weeks posttraining)	NA	NA

M, mean; SD, standard deviation; CR, cue reactivity; NA, no assessment; NS, no significant; NG, not given; ES, effect size; NES, no effect size; PANAS, Positive and Negative Affect Scale; DCS, D-cycloserine; VAS, Visual Analog Scale; MEM, memantine; PLAC, placebo; REACT, reactivation of smoking MMMs; MPSS, Mood and Physical Symptoms Scale; QSU-Brief, Brief version of the Questionnaire of Smoking Urges; MNWS, Minnesota Nicotine Withdrawal Scale; TNR, transdermal nicotine replacement; NicVAX, 3′-aminomethylnicotine *Pseudomonas aeruginosa* r-Exoprotein conjugate vaccine; CBT, cognitive behavioral therapy; CET, cue exposure therapy; ABM, Attentional Bias Modification; NRT, Nicotine replacement therapy. The symbols “↑, ↓” indicate increasing (↑) and decreasing (↓) respectively.

Notably, 7 of all the included articles only studied male smokers. In terms of age, all subjects were ≥ 18 years old and were generally categorized as youthful to middle-aged (20–50 years). Sample sizes for all studies ranged from 10 to 434, with follow-up ranging from 1 week to 6 months within 22 studies. Of the 67 included, only 6 had no measure of smoking cue-provoked craving, and the rest of the literature contained 29 articles that showed a significant reduction in smoking cue-induced craving, such as aripiprazole (97), baclofen (8), anodal transcranial direct current stimulation (tDCS) of the dorsolateral prefrontal cortex (DLPFC) (50, 100), repetitive transcranial magnetic stimulation (rTMS) (9), physical exercise (85–87, 93), olfactory stimuli (55) and 4-mg nicotine mini-lozenges (56). Twenty papers measured physiological parameters and 9 of them had significant differences between the intervention and the control groups. For example, vigorous exercise (54) reduces startle reflex amplitude, while varenicline (60, 107) reduces heart rate. There are 14 trails on brain function measurements, 11 of which are fMRI, the other 3 trails are EEG. All EEG measurements except LPP and N2 magnitude had statistically significant differences between the groups in P3. Functional MRI revealed brain activity mainly decreased in the medial orbitofrontal cortex (mOFC), ventromedial striatum (*VS*), ventromedial prefrontal cortex (vmPFC), ventral prefrontal cortex (vPFC), left anterior ventral insula (avInsula), nucleus accumbens (Nac) caudate, while increased in right DLPFC and brain default mode. The measures mentioned above are described in detail under each treatment topic below.

### Pharmacotherapy

The 28 included TUD-related pharmacotherapy studies, therapeutic agents were nicotine replacement therapy (NRT) which account for the largest proportion, at nearly 1/3, and the others were olanzapine, haloperidol, topiramate, divalproex, omega-3 fatty acids, intranasal oxytocin, propranolol, aripiprazole, bupropion SR, gemfibrozil, baclofen, and varenicline which make up the second proportion. Two of the studies were conducted on male subjects only, and 1 had no sex information. The male-to-female ratio of the remaining studies where approximately 1:2 to 3:1 see Table 1 for details.

For psychological indicators, 26 studies investigated the effect of drugs on cravings induced by smoking cues, resulting in about half of the studies finding no statistically significant differences between groups, while the other studies found that NRT (half of the included NRT-related studies, only one of them has ES which is 0.6 or 0.7, see Table 1 for detail), baclofen, olanzapine, varenicline, bupropion SR, omega-3 fatty acids and intranasal oxytocin (ES: η^2^_*p*_  =0.2) reduced cue-induced craving compared to the control group. For other varenicline-related studies, they all showed no statistically significant differences. Acute varenicline only selectively reduced tonic cravings rather than cue-induced cravings (46), which might be associated with different psychological processes. Divalproex and aripiprazole (light smokers with 10 mg) were reported to enhance cue-induced cravings (62). There is no statistically significant difference between the intervention and control groups in terms of smoking withdrawal symptoms (except that 4 mg nicotine lozenge attenuated it (ES = 0.37)) and affect.

Regarding physiological indicators, varenicline slowed HR but had no significant difference in muscle sympathetic nerve activity, baroreflex sensitivity and BP (107). In contrast, NRT, aripiprazole, propranolol, and gemfibrozil had no significant difference in HR, SC; HR, BP; HR, ST, SC; SC, HR and left corrugator electromyography, respectively.

In terms of brain function metrics, it has found that varenicline related to reduced brain activity of *VS* and mOFC under fMRI scan (45). Both varenicline and bupropion SR showed no difference in LPP amplitude before or after the intervention (48). It has also found that baclofen enhanced resting brain activation of the right DLPFC and decreased neural response in the vmPFC and left avInsula under fMRI scan (8). Interestingly, Novick and colleagues (82) found that there was not different in the effect of progesterone between males and females in the neural activation of ACC, posterior cingulate cortex (PCC), left lateral occipital cortex (LLOC), and left middle temporal gyrus (LMTG) under fMRI scan.

### Non-invasive brain stimulation

Of the 9 non-invasive brain stimulation trials included, the two main interventions were tDCS, and rTMS see Table 2 for details. Of these, 4 were tDCS and 3 (2 studies’ stimulated site was the left DLPFC (50, 99) and 1 study’s stimulated site was the left and right DLPFC (100)) of which reduced cue-induced craving while the other one (bilateral cathodal stimulation of the FPT area or cathodal over right FPT (98)) did not assess this indicator, and 5 were rTMS and 3 (2 both stimulated the left DLPFC (64, 65) and 1 bilaterally stimulated neuronal pathways in the lateral prefrontal cortex and insula (9)) of which reduced cue-induced craving. A multicentre, double-blind RCT (9) found that rTMS reduced cue-induced craving, which led to the first clearance by FDA for rTMS as an aid in smoking cessation for adults. Although one session of active rTMS over the left DLPFC did not reduce cue-induced craving, it still reduced blood oxygen level-dependent (BOLD) activation in contralateral mOFC and ipsilateral NAc under pre-and post-intervention fMRI scans. One study reported that tDCS reduced smokers’ craving (ES: *d* = 0.410) by increasing the coupling between DLPFC and parahippocampal gyrus (ES: *d* = 0.589) (99).

### Psychotherapy

Of the 11 psychotherapies included, 3 were mindfulness-related interventions, 3 was neurofeedback, 1 was attentional bias modification (ABM), 1 was retrieval-extinction training, 1 was virtual reality cue exposure (VRCE), 1 was augmented reality cue exposure (ARCE), and 1 was stress-based intervention. The subjects of two of the psychotherapy-related studies were both males (see Table 3 for details).

Regarding psychological indicators, compared to the control group, there were no statistically significant differences in mindfulness-related interventions, retrieval-extinction training, VRCE, ARCE and stress-based intervention in cue-induced cravings, while neurofeedback met with mixed results. As for other kinds of psychological indicators, compared to the control group, ABM, retrieval-extinction training, and a brief mindfulness-meditation intervention showed no difference in cognitive biases, negative effect, as well as error rates and reaction times on the smoking Go/NoGo, respectively.

For physiological indicators, retrieval-extinction training and stress-based intervention had nonsignificant difference in HR, BP and HR, BP, SC, respectively. The other psychotherapy-related studies had no measure of physiological indicators.

Under fMRI scan, Mindfulness-Oriented Recovery Enhancement was demonstrated that the decrease in cue-reactivity BOLD (CR-BOLD) response in the *VS* (ES: *d* = 1.57) and vPFC (ES: *d* = 1.7) and the increase in positive emotion regulation BOLD (ER-BOLD) response, as well as the increase in resting-state functional connectivity (rsFC) between rACC and OFC. These manifestations may be related to the facilitation of the reorganization of reward processes, suggesting that they may play a role in the pathophysiology of nicotine addiction (53). Under fMRI scan, neurofeedback has been shown to improve neural activity and functional connectivity between target regions of interest (ROIs; ROIs1: ACC and medial pFC, ROIs2: PCC and precuneus) (106) and reduced craving-related prefrontal cortex (PFC) activation (66). On EEG, a brief mindfulness-meditation intervention reduced P3 amplitude without significant effects on N2 amplitude during the task of NoGo vs. Go (105). Another finding was on neurofeedback training which reduced P300 amplitude with moderate effect size (*d* = 0.64) (7).

### Exercise therapy

Of the 6 exercise therapies included, 5 (2 of them have effect size in the range of 0.4–2, see Table 4 for details) of them found that exercise therapy could significantly reduce smoking cue-elicited craving compared with control group, while light and vigorous intensity aerobic exercise had no significant effect on it but reduced startle reflex magnitude in vigorous exercise (54) (see Table 4 for details). In addition, it was found that a 15-min exercise could attenuate withdraw symptoms and attentional biases (85, 87, 93). For neuroimaging indicators, 10 min moderate-intensity stationary cycling was found to activate brain default mode (Broadmanns Area 10) (86).

### Other therapies

Of the 13 other tobacco cessation treatments included, 10 were combination treatments, 1 was vaccine (NicVAX), 1 was acute tobacco smoking, and 1 was olfactory stimuli. Among the included studies, 1 (89) found no information on gender (see Table 5 for details). Regarding psychological indicators, olfactory stimuli, either a pleasant or unpleasant odor, reduced cue-evoked craving (55). Interestingly, compared to the control group, over half of combination treatment studies and acute tobacco smoking found no statistically significant differences in cue-induced craving between the groups for either cessation seekers or unmotivated quitters while about half of the combination treatment studies found the treatments reduced craving. As for withdrawal symptoms and attentional bias, they were all showed mixed results in the certain combination treatments. For neuroimaging indicators, only Havermans et al. (104) assessed this indicator and found that NicVAX did not modulate brain activity to smoking cues. Regarding physiological indicators, combination treatments-related studies were inconsistent with each other on SC and HR. And there were no significant differences between groups in BP, heart rate variability, ST, and left corrugator electromyogram, whereas it was found that naltrexone combined with transdermal nicotine replacement could increase mean arterial pressure.

### Cue-reactivity paradigms

The cue-reactivity paradigms in the 67 included articles were essentially composed of smoking cues and neutral cues, with 2 (48, 62) combining pleasant and unpleasant picture cues in Table 6 for details. Thirty-one trials based on vision (*in vitro* cues), 20 trials based on behavior (*in vivo* cues), 8 trials based on behavior and vision (*in vivo*/vitro cues), 2 trials based on behavior (*in vitro* cues), 2 trials based on vision and auditory (*in vitro* cues), 1 trial based on vision (*in vivo* cues), 1 trial based on behavior, auditory and vision (*in vivo*/vitro cues), 1 trial based on behavior and vision (*in vivo*/vitro cues), and 1 trial based on behavior (*in vivo*/vitro cues; see Supplementary Table S3).

**Table 6:** Smoking cue reactivity paradigms.

Authors (year)	Stimulus material	Types	Description
Hutchison et al. (1999) (69)	Cigarette	Behavior (*in vivo* cues)	Participants were then provided with a cigarette of their preferred brand, a lighter, and an ashtray and were instructed to light and hold the cigarette without taking a puff. The participants held the cigarette for 60 s before extinguishing it.
Sayette et al. (1999) (55)	Cigarette	Behavior (*in vivo* cues)	A tray holding an inverted plastic bowl was placed on the participants’ desk. This bowl covered cigarettes, an ashtray, and a lighter. From the control room, the experimenter instructed participants to remove the cover, light the cigarette without putting it in their mouths, and stare at it for 10s. After 10s, participants verbally rated their urge to smoke on the 0–100 scale. Participants were next instructed to extinguish the cigarette.
Tiffany et al. (2000) (70)	Cigarette, imagery	Behavior (*in vivo*/vitro cues)	On imagery trials, scripts were presented over headphones. Participants had their eyes closed throughout the imagery procedure. The three cigarette imagery scripts contained explicit craving descriptors, and each included descriptions of watching people smoke to provide overlap with the primary stimulus content of the cigarette *in vivo* trials. The three neutral imagery scripts were devoid of any craving or smoking content. During the six *in vivo* trials, the participant opened his or her eyes when cued by a tone presented over the headphones. The participant then observed a same-gender experimenter, seated 10 ft. (3 m) away, either lighting and smoking the participant’s brand of cigarettes or pouring a glass of water and drinking from the glass. At the end of the cue-exposure period, the participant was signaled to close his or her eyes and think about what he or she had observed until hearing the word *stop*. The sequence of events for the *in vivo* trials paralleled the imagery trial sequence: 30 s of baseline, 50 s of cue exposure, 30 s of thinking about the cue presentation, and 30 s of relaxation.
Shiffman et al. (2003) (71)	Cigarette	Behavior (*in vivo* cues)	After 5 min of adaptation, the cue-exposure manipulation was begun. Subjects were instructed to lift up an opaque bowl, under which had been placed an unopened pack of their favored brand of cigarette, two lighters (one as back-up), and an ashtray. They were then instructed to: (1) unwrap and open their pack of cigarettes; (2) remove one cigarette; (3) hold the cigarette in their hand and light it without placing it in their mouth; and (4) hold the lit cigarette directly in front of them without smoking it. These procedures were standardized and designed to take 30 s. Participants were instructed to look at the lit cigarette for 60 s and then extinguish it in the ashtray.
Hutchison et al. (2004) (57)	Cigarette	Behavior (*in vivo* cues)	Participants were first exposed to control cues by asking them to hold a pencil for 3 min. The exposure to the control cue was followed by an assessment of craving and a 5-min interval prior to exposure to the smoking cue. Exposure to the smoking cue consisted of instructing the participants to remove one of their preferred brand of cigarettes from a pack and light it without putting it in their mouths by holding it in the flame for several seconds. Participants were then instructed to focus their attention on the lit cigarette.
Waters et al. (2004) (72)	Cigarette	Behavior (*in vivo* cues)	Participants were seated at a comfortable distance from the computer. On the desk was a small box that, unbeknownst to the participants, contained a recently opened box of their preferred brand of cigarettes. After about 6 min of preexposure testing, participants were instructed to open the box, take out one of the cigarettes, and look at it for a few seconds. They were then instructed to hold the cigarette in their smoking hand, in the manner they would if they were between puffs when smoking, and to continue to look at it. Next, they were instructed to put the cigarette back into the box and to close the lid.
Mahler et al. (2005) (58)	Cigarette	Behavior (*in vivo* cues)	Subjects were exposed to smoking cues and to neutral cues. These cues were presented in two distinctive rooms, separate from the waiting room where the subjects spent most of the sessions. The smoking cue consisted of a lit cigarette held, but not smoked, by the subject. The neutral cue consisted of a pencil cut to the same length as a cigarette. Subjects held the cues for 4–7 min, until they completed their subjective reports and the reaction time task.
Morissette et al. (2005) (73)	Imagery	Behavior (*in vitro* cues)	Participants listened to and imagined each of the scripts with their eyes closed. For demonstration purposes, a practice script was first presented. Four types of experimental imagery scripts were then presented: (a) anxiety plus smoking cues, (b) anxiety cues alone, (c) smoking cues alone, and (d) neutral cues. Two scripts of each type were used, totaling eight imaginal scenarios. Scripts were counterbalanced for both order and sequence. Each script sequence consisted of a 30-s baseline period, 50-s script presentation period, and 30 s of active imagery by the participant terminated with the word “stop.” Participants were then asked to open their eyes and complete postexposure trial questionnaires asking them about how they felt during the most recent scenario.
Niaura et al. (2005) (74)	Cigarette	Behavior (*in vivo* cues)	After 5 min of adaptation, and a second pre-cue craving assessment, the cue-exposure manipulation was begun. Subjects were instructed to lift up an opaque bowl to expose an unopened pack of their favored brand of cigarette, a lighter and an ashtray. They were then instructed to open the pack of cigarettes, remove a cigarette, light it without placing it in their mouth, and hold the lit cigarette directly in front of them without smoking it. These procedures were standardized and designed to take 30 s. Participants were instructed to look at the lit cigarette for 60 s and then extinguish it in the ashtray.
Reid et al. (2007) (59)	Cigarette, video	Behavior and vision (*in vivo*/vitro cues)	During the cigarette cue session, each patient was presented with a lighter, ashtray, and three to four new packs of cigarettes, including one of their preferred brand. All items were placed on the table in front of them. Initially, the patients handled each of the packs and then selected their preferred brand as if they were about to smoke. Then, the research assistant opened the selected pack, and the patient, using his/her non-writing hand, removed a cigarette and held it in his/her hand, smelled the tobacco in the cigarette, and lit the cigarette (with the aid of a research assistant so as not to inhale smoke). The lit cigarette was then placed in the ashtray in front of the patient, who viewed the lit cigarette and smelled the smoke for approximately 30 s before extinguishing it in the ashtray (paraphernalia phase lapsed time, 5 min). The patient then watched a video depicting scenes of people smoking: restaurant/bar with two people smoking cigarettes, a person having a cigarette after completing a meal, co-workers having a cigarette break outside an office building, and people speaking about the pleasures of smoking (video lapsed time, 5 min). After the video, the patient re-lit the cigarette in the ashtray and smelled the smoke of the burning cigarette for another 20–30 s.
Rohsenow et al. (2007) (75)	Cigarette	Behavior (*in vivo* cues)	During a 4-min neutral cue trial, a tray with a pencil, eraser, and small pad of paper were placed on the table, and participants were asked to hold and look at the pencil and eraser throughout the 4 min, following which they covered the cues, and the self-report measures were completed. Three 4-min smoking cue trials followed. A tray containing a pack of the participant’s own brand of cigarettes, a clean ashtray, and a lighter was brought in. Participants were asked to take a cigarette out of the pack and hold it for 2 min, then light it without putting it in their mouth, and look at the lit cigarette for the next 2 min. At the end of 4 min, the participant extinguished the cigarette, covered the cues, completed the self-report measures, and had the smoking materials removed.
Taylor et al. (2007) (85)	Cigarette	Behavior (*in vivo* cues)	Participants were required to watch the lighting of a cigarette (one of their favorite brands) that was placed in front of them. They were asked to hold the cigarette between their fingers but were not allowed to smoke it.
Fregni et al. (2008) (100)	Cigarette, video	Behavior and vision (*in vivo*/vitro cues)	For the cigarette manipulation cue, subjects were instructed to open a pack of their favored brand of cigarette, pick up a cigarette, place it in their mouths, pick up a lighter, and pretend to light and smoke the cigarette. These procedures were standardized to be performed in 30 s. Subjects were then asked to put the cigarette away and were shown a movie of 5 min’ duration presenting people smoking in a pleasant way. (Six different equivalent movies were randomized across subjects, as the subjects were exposed to a different movie before and after the 3 types of treatment).
Boggio et al. (2009) (50)	Cigarette, video	Behavior and vision (*in vivo*/vitro cues)	The same as Fregni et al. (100).
Bowen et al. (2009) (52)	Cigarette	Behavior (*in vivo* cues)	The cue exposure trial was delivered in four stages, each stage lasting approximately 4–6 min, and each with increasing levels of intensity. Audio recordings instructed participants to first open a pack of cigarettes (stage 1), place a cigarette on the table in front of them (stage 2), place cigarette in their mouth (stage 3), and bring a lighter to the cigarette without igniting the cigarette (stage 4).
Liu et al. (2009) (97)	Cigarette	Behavior (*in vivo* cues)	Cues consisted of physical objects presented simultaneously to the participants in two 3-min sessions, during which participants were asked to handle each item. During the neutral cue session, a pen, a post-it pad, and a mixture of spices (e.g., cinnamon, *Illicium verum*) were presented. The pen and post-it pad were chosen because they resembled a cigarette and lighter, while the spices presented an olfactory cue comparable to the smell of cigarettes. During the cigarette cue session, cigarettes of participants’ preferred brand, a lighter and an ashtray were used as cigarette cues. Participants were asked to light the cigarette and perform their normal smoking behaviors (including smelling the cigarette and handling the cigarette and lighter) except for inhaling the smoke.
Janse Van Rensburg et al. (2009a) (86)	Image	Vision (*in vitro* cues)	The 60 images (smoking and neutral images) were randomly presented in each scanning session and for each participant using E-prime software. Images were viewed on a screen placed at the foot of the scanner via a mirror mounted on the head coil. Each image was presented for 3 s. A button, placed in each of the participant’s hands, was pressed upon presentation of the image to ensure attentional focus. The button-press for smoking or neutral images was randomized for hand dominance between participants. After each image, a white screen with a black fixation cross was presented for a randomly determined period of 8, 10, or 12 s, and participants were asked to view this between smoking images to remain focused. The duration of image presentation and the inter-stimulus-interval chosen fall between those reported in other event-related studies in this area.
Janse Van Rensburg et al. (2009b) (87)	Image	Vision (*in vitro* cues)	Participants were required to passively view a series of matched-paired smoking (e.g., hand holding cigarette) and neutral (e.g., hand holding pen) images presented with a custom written C-software program using the Eyelink programmers’ function library.
Santa Ana et al. (2009) (76)	Cigarette	Behavior (*in vivo* cues)	Participants were presented with a covered tray containing a pack of their favored brand of cigarettes, a lighter, and an ashtray. Following the instruction to remove the cover and look at the smoking objects in the tray, participants were asked to provide an initial urge-to-smoke rating (conducted at 0 s) on a 10-point Likert scale. Participants were instructed to remove a cigarette from the pack and hold the cigarette the way they normally would using their dominant hand when smoking, after which time skin conductance measures were assessed using the non-dominant hand for 5 min. After requesting participants to smell the cigarette, they were instructed to place the cigarette in the ashtray, flick the lighter until they saw the flame, put down the lighter and hold the cigarette again. At 8 and 38 s, participants were again requested to rate their urge-to-smoke on the 10-point Likert scale. At 45 s, participants were requested to rehearse the strategy of urge-surfing (e.g., coping with the urge-to-smoke) while they were holding the cigarette in their hand. At 75 s, participants were requested to place the cigarette in the ashtray, at which time they were requested to practice relaxation, visualization as a non-smoker, and coping skills for smoking triggers until the end of the skin conductance assessment. Altogether, participants received a series of six smoking cue exposure trials provided at 30–40 min intervals within each of the two experimental sessions.
Hussain et al. (2010) (91)	Image	Vision (*in vitro* cues)	Fifty minutes after smoking, subjects viewed a block of neutral pictures then a block of smoking-related pictures. Each block presented 10 pictures without pause (6 s/picture).
Brandon et al. (2011) (60)	Image	Vision (*in vitro* cues)	A total of 24 pictures with 12 pictures from each category (smoking and neutral) were presented randomly during each session. Different sets of pictures were used across the three assessment sessions to minimize habituation. Pictures were displayed for 6 s each on a 20″ computer monitor located 2.5 ft. in front of participants, controlled by software that synchronized cue presentations with physiological data collection.
Culbertson et al. (2011) (61)	Video	Vision (*in vitro* cues)	Each functional Magnetic Resonance Imaging (fMRI)scanning session consisted of 3 runs, with each run including 3 cue conditions. During each run, participants viewed 1 neutral cue video, 1 crave-allow cigarette-related cue video, and 1 crave-resist cigarette-related cue video. Prior to initiation, participants were instructed to allow themselves to crave cigarettes during the cigarette-related cue videos unless explicitly in structed to resist craving (eg, “during the next video clip, try to resist any feelings of craving for cigarettes”). The cue videos were presented in a randomized fashion (Latin square design).
Elibero et al. (2011) (68)	Image	Vision (*in vitro* cues)	Participants viewed a randomized sequence of 12 smoking-related and 12 neutral images on a 20-inch computer monitor. The sequence for each of the 24 images consisted of a 2-s baseline period, a 6-s picture-viewing period, followed by a subjective craving rating obtained via computer (0–20 scale). A variable 12–20 s intertrial interval separated the end of each rating period from the start of the next trial.
Franklin et al. (2011) (45)	Video	Vision (*in vitro* cues)	Images were acquired during a scanning session that included, in sequence, a 1-min localizer scan, a 5-min continuous arterial spin-labeled (CASL) resting-baseline scan, a 10-min nonsmoking cue CASL scan, a 5-min high-resolution structural scan, and a 10-min smoking cue CASL scan. Nonsmoking cues were shown before smoking cue videos to minimize interference in “carryover” arousal initiated when drug cues are shown first, which can potentially affect responses to nondrug cues.
Ditre et al. (2012) (62)	Image	Vision (*in vitro* cues)	An established picture-viewing paradigm was adapted to assess subjective, behavioral, and physiological responses to 30 affective pictures (10 pleasant, 10 unpleasant, and 10 neutral) and 10 smoking-related pictures. Participants were instructed to view each slide on a large screen located approximately 2.5 m directly in front of them, and to watch each slide for the duration of its appearance. Each picture was presented for 6 s. After the slide series had been viewed once, the experimenter informed participants that they would view the same series of slides again, to view each slide for as long as they wished (maximum of 20 s), and to press a joystick button to turn off the slide before making ratings. Duration of viewing time was measured to the nearest millisecond to provide a behavioral measure of interest.
Kamboj et al. (2012) (88)	Imaginal cues, cigarette, video	Behavior and vision (*in vivo*/vitro cues)	The standardized exposure/response prevention (Exp/RP) procedure involved sequential presentation of the three types of cue, starting with imaginal cues (participants were guided through a vivid re-imagining of the two craving scenarios elicited as above; 5–6 min), followed by *in vivo* (participants handled their preferred cigarettes and lighter as if preparing to smoke but without bringing the cigarette close to their mouths; 2 min) and video of a solitary man smoking while facing the viewer (2 min).
Hitsman et al. (2013) (46)	Cigarette	Behavior (*in vivo* cues)	During each cue session of approximately 60 min, participants were exposed to a smoking cue and a neutral cue in a randomly assigned sequence of either smoking-neutral or neutral-smoking. The sequence was balanced and preserved for each participant’s second treatment session. Each cue exposure lasted for 1 min and was preceded by a 5 min adaptation period (i.e., participants were instructed to rest for 5 min) during a 6-min block of time. In the smoking cue condition, participants lit and held their preferred brand of cigarette and then extinguished it. In the neutral cue condition, participants sharpened a pencil and then held it.
Janse Van Rensburg et al. (2013) (54)	Cigarette	Behavior (*in vivo* cues)	Participants were presented with a neutral cue (a roll of tape and stapler) for 60 s, and then a smoking cue (asked to hold their own brand lit cigarette) for 60 s.
Li et al. (2013) (64)	Image	Vision (*in vitro* cues)	Seventy scenic images (e.g., mountains), 40 neutral control images (e.g., a person holds a pen), and 40 cigarette-smoking cue images (e.g., a person lighting a cigarette) were presented in four blocks: #1 scenic images–5 min, #2 neutral control images–1.5 min, #3 scenic images–5 min, and #4 cigarette-smoking cue images–1.5 min. After 15 min of real or sham transcranial magnetic stimulation (rTMS), participants viewed the images again and rated their cravings.
Du et al. (2014) (77)	Cigarette	Behavior (*in vivo* cues)	The same as Shiffman et al. (71).
Fong et al. (2014) (93)	Cigarette	Behavior (*in vivo* cues)	Researchers asked participants to place a cigarette of their preferred brand on the desk in plain sight.
Havermans et al. (2014) (104)	Image	Vision (*in vitro* cues)	Participants were presented with 20 blocks of three color photographs with smoking-related or neutral content. The smoking-related images (*n* = 30) included the heads and mouths of people smoking, hands holding a cigarette and cigarettes in ashtrays or in a pack. Neutral images (*n* = 30) were matched for shape of the object and general content and included for example a person brushing his teeth, a hand holding a screwdriver, chopsticks on a bowl and pencils in a pack. Each stimulus was presented for 6 s 18 s per set. Stimulus sets were presented in random order with intervals of 18 s, during which a fixation cross was visible.
Meng et al. (2014) (98)	Image	Vision (*in vitro* cues)	The tests started with the appearance of a fixation dot in the center of the screen. After 2 s, a our-quadrant picture was presented on the screen. Each picture consisted of 4 different objects with one in each quadrant. One of the objects was smoking or cigarette related cue (e.g., a burning cigarette), the others were neutral stimuli (e.g., a cup). The location of the smoking cue in the four quadrants was randomized and counterbalanced. The presentation time of the visual stimuli was 5 s with a 5e10 s time out. During the presentation of visual stimuli, participants could explore the screen freely.
Rabinovitz et al. (2014) (102)	Image	Vision (*in vitro* cues)	participants were asked to view 14 full 14-inch screen pictures depicting photographic cigarette-related cues (e.g., hands holding lit cigarettes, smoking-related objects and people smoking cigarettes). Cues were presented for 3 s in random order, each picture was presented twice. Target cues (*n* = 7) were pictures of animals. Participants were asked to press a button whenever they saw a target. Total presentation time took about 1.5 min, depending on individual reaction times.
Schlagintweit et al. (2014) (94)	Video	Vision (*in vitro* cues)	Participants were comfortably seated at a desk, in front of a computer monitor, and were instructed to view two 2 min video clips that depicted neutral and smoking cues. The first clip, a neutral cue, depicted various individuals getting haircuts. The second video was a smoking cue, consisting of various individuals smoking cigarettes.
Begh et al. (2015) (90)	Image	Vision (*in vitro* cues)	Attentional bias was assessed using the visual probe and pictorial Stroop task: eighteen picture pairs of smoking-related and neutral pictures were used across attentional bias assessment and training tasks. The visual probe assessment comprised 192 trials presented in two blocks, with each picture pair presented for 500 ms. Eight practice trials with neutral picture pairs were presented before the first assessment block. Presentation of the picture pairs and probes were counterbalanced, i.e., each permutation of picture pair and probe type was presented within each block. Thus, each type of probe appeared in the location of the smoking-related and neutral picture with equal frequency. Each block of trials was presented in a new random order for each participant, using EPrime version 2.
Das et al. (2015) (89)	Image	Vision (*in vitro* cues)	The task used two types of image pairs: smoking pictures paired with composition-matched neutral images (n = 20) or control neutral-neutral (*n* = 20) pairs. Image pairs appeared for 500 or 2000 ms and were replaced by probes either contralateral or ipsilateral to the target (smoking-related). Trial presentation was counterbalanced for duration, target side and probe/target congruence.
Kim et al. (2015) (106)	Video	Vision (*in vitro* cues)	Each rtfMRI-NF run lasted 258 s and consisted of the following: (1) a calibration period to align the coordinates of an MR-compatible eye tracker, (2) a period of fixation to a white cross on a black screen, (3) presentation of the “ready” command, (4) presentation of a video clip showing smoking (i.e., the rtfMRI-NF period), (5) a period for subjective ratings of cigarette cravings, and (6) a fixation period at the end of the scan. Twelve video clips (each of 3-min duration) containing male smokers lighting and smoking a cigarette were collected via an Internet search.
Pachas et al. (2015) (47)	Imagery	Behavior (*in vitro* cues)	To establish baseline parameters, participants listened, through headphones, to a relaxation script, heart rate, skin conductance, left corrugator electromyogram were measured and subjects completed assessments of baseline emotional state and craving. This procedure was then repeated with two counter-balanced personalized smoking and two standard neutral scripts, 40 s each, beginning with a neutral script. Each script presentation consisted of four sequential 30-s periods: baseline, read, imagery, and recovery. Subjects were instructed to listen carefully during the playing of the scripts and to attempt to imagine as vividly as possible each experience as it was presented (read period) and, on script termination, to continue to imagine the experience from beginning to end (imagery period) until they heard a tone. They were further instructed to stop imagining the script at the tone and to relax (recovery period) until a second tone was heard.
Elfeddali et al. (2016) (103)	Image	Vision (*in vitro* cues)	Eight sets of 12 matched smoking-related [e.g., smoking people, cigarette (packages), etc.] and neutral [e.g., nonsmoking people, pencils (packages), etc.] picture pairs for the Visual Probe Task.
Haarmann et al. (2016) (107)	Cigarette	Behavior (*in vivo* cues)	Participants were allowed to touch a pack of their favorite cigarette brand and their lighter for two minutes.
Hartwell et al. (2016) (66)	Image	Vision (*in vitro* cues)	Each rtfMRI scanning visit consisted of 4 10-min smoking cue exposure runs: an initial craving region of interest (ROI) identification run (run 1) followed by 3 neurofeedback runs (runs 2–4). Images were presented with E-Prime 2.0 software and viewed via a mirror attached to the head coil. Each run was composed of a smoking cue exposure task used in previous studies with 3 types of blocks: smoking-related pictures (smoke), non-smoking related pictures (neutral) and a crosshair (rest). Each block consisted of 5 pictures displayed for 4.4 s each.
Miller et al. (2016) (63)	Cigarette, image	Behavior and vision (*in vivo*/vitro cues)	Cue exposure consisted of viewing a 5-min set of pictures presented for 6 s each, with the pictures consisting of images of cigarettes, smoking paraphernalia, and individuals or groups of individuals smoking. Participants viewed a different set of images at each session. Immediately after viewing the images, participants were asked to light a cigarette provided by the researchers (Marlboro Light brand) and hold the cigarette for five minutes without smoking it.
Schlagintweit et al. (2016) (95)	Video	Vision (*in vitro* cues)	Two 2-min video clips depicting individuals getting haircuts (neutral cue) and smoking cigarettes (smoking cue) were used to assess cue-induced cigarette craving. The neutral cue was presented prior to the smoking cue to prevent carryover effects, and video clips were presented within five minutes of one another in order to minimize the possibility of changes in craving resulting from the passage of time.
Froeliger et al. (2017) (53)	Image	Vision (*in vitro* cues)	The cue reactivity task presented alternating blocks of control images (e.g., pencil) (40 s), followed by a fixation and a craving rating response screen (30 s), and then smoking-related images (e.g., cigarette) (40 s) over the course of 8.5 min.
Germeroth et al. (2017) (67)	Video, image	Vision (*in vivo*/vitro cues)	The cue-reactivity assessments involving the presentation of familiar smoking video cues and novel smoking picture cues. The video had smoking content for the retrieval-extinction [R-E] group but neutral, nonsmoking content for the nonsmoking related retrieval–extinction [NR-E] group, and postextinction cues (all cues contained smoking content) during the R-E or NR-E training sessions. Only the 2 R-E or NR-E training sessions involved a retrieval-cue presentation (5-min smoking retrieval videos for the R-E group and neutral videos for the NR-E group) during the cue-reactivity assessment.
Jones et al. (2017) (78)	Cigarette	Behavior (*in vivo* cues)	Two opaque pitchers were placed on the participant’s desk at the beginning of the session. Hidden under one pitcher was a glass and a bottle of spring water. An unopened pack of their favorite brand of cigarettes, lighter, matches, and an ashtray were hidden under the second pitcher. During the cue session, participants were first shown the water bottle and asked to look at, hold and sniff it, and take a drink of the water inside. After a 5-min relaxation period, participants were visually exposed to the smoking cues. A research nurse subsequently instructed participants to open their pack of cigarettes and take one out. They were then instructed to hold the cigarette in their mouth, then hold it in their hand, light it up without placing in their mouth, hold it directly in front of them without smoking, and then finally extinguish it in the ashtray.
Li et al. (2017) (109)	Video, image	Vision (*in vitro* cues)	The smoking cues consisted of a house made smoking cue video, and a series of smoking related images. The smoking related images included the heads and mouths of people smoking, hands holding a cigarette, and cigarettes in ashtrays or in a pack during rTMS. Cue Presentation and Craving Measurements in the Scanner–Visual stimuli were adapted from previous smoking cue fMRI studies conducted by our group, and were presented in a block design using standardized pictures. The pictures consisted of, people smoking or engaged in matched neutral activities, and objects related to smoking (cigarettes, ashtrays, etc.) or matched neutral objects (pencils, dishes, etc.).
Yang et al. (2017) (99)	Image	Vision (*in vitro* cues)	In each trial, a picture with either a smoking related stimuli or a neutral stimuli was presented for 900 ms following a fixation cross (jittered from 1,100 ms to 5,100 ms). Two to five semi-randomly distributed lines were displayed within each picture. Participants were instructed to count the number of lines and to press the corresponding button as fast as possible. The picture content was not related to the number of lines. The task was composed of 150 trials.
Andreu et al. (2018) (105)	Image	Vision (*in vitro* cues)	A smoking Go/NoGo task: a series of smoking or neutral pictures were presented. Each picture was displayed for 200 ms and had a blue or yellow frame. Frame color indicated whether a stimulus was a Go or NoGo trial. Each stimulus was followed by a black screen for a randomly varying duration between 1,000 ms and 1,500 ms. Participants completed the task in two blocks of 240 trials each, one with smoking pictures and one with neutral pictures. Block order was randomized and in the middle of the task an additional block with 18 emotionally positive pictures was used to washout possible carry-over effects.
Brandon et al. (2018) (79)	Image	Vision (*in vitro* cues)	12 smoking related and 12 neutral control images were randomly presented to each participant while craving measures were obtained. Smoking cues included photos that have elicited substantial craving reports in our prior research. Neutral cues consisted of pictures from the International Affective Picture System, and included objects, people, and situations that have been rated as neither pleasant, unpleasant, or arousing. Following picture offset, smoking craving ratings were obtained on a visual-analog scale.
Gendy et al. (2018) (92)	Cigarette	Behavior (*in vivo* cues)	The smoking cue was a pack of cigarettes and a lighter. Participants were instructed to light the cigarette without puffing and hold it for 30 s while the physiological recordings were measured. Then the participant was asked to extinguish the cigarette. The neutral cue was an unsharpened pencil, a notepad, and a sharpener. Participants were instructed to sharpen the pencil and hold it as if writing for 30 s.
Nides et al. (2018) (56)	Cigarette	Behavior (*in vivo* cues)	Each participant was left alone in a well-lit, temperature-controlled, well-ventilated, sound-attenuated room and received study instructions via audio-recording. After a 5-min acclimatization period, participants opened an opaque box containing a pack of their first or second choice brand of cigarettes, 2 cigarette lighters, and an ashtray. They were then instructed to unwrap and open the pack of cigarettes, remove one cigarette, hold it in their hand, light it without placing it in their mouth, and hold the lit cigarette directly in front of them without smoking it. Next, they were instructed to look at the lit cigarette for 60 s before extinguishing it in the ashtray.
Bu et al. (2019) (7)	Image	Vision (*in vitro* cues)	There were 330 pictures [150 smoking-related (e.g., a cigarette in the hand), 150 neutral (e.g., a pencil in the hand), and 30 animal-related (e.g., a kangaroo) cues] selected from our previous studies. These pictures were divided into six blocks, including three smoking blocks and three neutral blocks. The block design helped improve the signal-to-noise ratio of EEG smoking cue reactivity and was consistent with the later neurofeedback training design. The order of six blocks was made random across participants. Within a block, each trial contained a picture presented for 1.5 s and a fixation (+) was presented for 1–1.5 s. Animal pictures were shown randomly during all blocks. After completing a block, participants had a 90-s rest.
Otto et al. (2019) (81)	Image, imaginary, cigarette	Behavior, auditory and vision (*in vivo*/vitro cues)	The cue exposure therapy had three components: exposure to slides of smoking (visual) exposure to emotions and imagined situations that most reliably triggered an urge to smoke (emotional/imaginal), and exposure to a participant’s own cigarettes and pack (*in vivo*).
Versace et al. (2019) (48)	Image	Vision (*in vitro* cues)	Participants were shown one of three equivalent picture sets. Each set included 96 pictures: 24 pleasant (8 erotica, 8 romantic, 8 pleasant objects), 24 unpleasant (8 mutilations, 8 sad, 8 unpleasant objects), 24 neutral, and 24 cigarette-related images. The images were selected from the international affective picture system and from other sets used in previous studies. At each visit, participants saw a different picture set and the order of presentation was randomized across participants. At each visit, the frequency of each order presentation was similar across the three medication groups. Participants viewed the picture slideshow on a plasma television screen at a viewing distance of approximately 1.5 m. E-Prime software, running on a Pentium 4 computer, controlled the picture presentation.
Ketcherside et al. (2020) (8)	Video, cigarette	Behavior and vision (*in vivo*/vitro cues)	The smoking cue (SC) videos featured actors smoking, while using language explicitly designed to induce desire for a cigarette (e.g., “The cigarette I enjoy most is the first cigarette of the day”). The non-SC videos featured actors, but they were not smoking, and instead, told short stories unrelated to smoking and without smoking reminders. During the SC videos, subjects held one of their own cigarettes in their preferred hand, and a match was lit and extinguished, providing visual and olfactory stimuli to enhance neurophysiological and subjective cue reactivity. During the non-SC video, subjects held a freshly sharpened pencil.
Kotlyar et al. (2020) (80)	Virtual reality (VR)	Vision and auditory (*in vitro* cues)	The VR visor was placed immediately prior to the start of the cue presentation procedure and participants then proceeded through four virtual “rooms.” The first and last of these rooms had neutral cues (a TV displaying wildlife images) and the middle two had smoking cues. In one of the smoking cue rooms, participants navigated around a room containing a variety of objects commonly associated with smoking such as cigarette packs, ash trays, and burning cigarettes. The other smoking cue room contained people smoking, talking about smoking, and drinking. The sensations were primarily visual with some auditory input that included a voice-over providing information regarding the wildlife images displayed in the neutral rooms, and music and/or virtual people speaking in the cue rooms.
Li et al. (2020) (65)	Cigarette,video	Behavior and vision (*in vivo*/vitro cues)	We used structured 1.5 min exposure and interactions with real-life smoking paraphernalia (cigarettes, ashtray, lighter) immediately before each rTMS session. While rTMS was administered, subjects watched 15-min smoking cued video (scenes of individuals smoking in various environments) displayed on an iPad placed on a tripod at the foot of the treatment chair.
Lawson et al. (2021) (49)	Cigarette	Vision (*in vivo* cues)	On each of multiple Choice Behavior Under Cued Conditions (CBUCC) trials, participants are exposed to an *in vivo* cue (e.g., a lit cigarette, a cup of water). After rating craving in the presence of the cue, the participant spends real money ($0.01 to $0.25) to gain access to the cue; the more the participant spends, the greater the probability that the door will be unlocked and the cue can be sampled on that trial (probabilities range from 5 to 95%).
Zangen et al. (2021) (9)	Imaginary, audio, image	Behavior and vision (*in vivo*/vitro cues)	Each repetitive rTMS session was preceded by a 5-min provocation procedure, which included participants imagining their greatest trigger for craving, listening to an audio script with instructions to handle a cigarette and a lighter, and viewing pictures of smoking.
Malbos et al. (2022) (108)	Virtual reality	Vision and auditory (*in vitro* cues)	virtual environments (VEs) offer distinct craving-inducing scenarios: having a drink with people smoking in a virtual beach bar at sunset; walking with avatars smoking on the terrace of a restaurant; being in a furnished living room or its balcony with a beer, an ashtray and a lighted cigarette; waiting at a bus stop with avatars smoking around; taking a break in a workplace with smoker colleagues and driving a virtual car on a road during a traffic jam. During exposure, the investigator can trigger specific events within the VE (i.e., avatars talking about smoking or inviting the participants to smoke a cigarette or drink a cup of coffee). These options allow for progressive increases in the intensity of induced craving to modulate the degree of exposure at various times. Dynamic VEs also provide the participant with direct, realistic interactions (such as opening doors, virtual human interactions, grabbing objects and physical or mechanical reactions to the user’s presence).
Marques et al. (2022) (101)	Image	Vision (*in vitro* cues)	A computer-based paradigm was developed with OpenSesame v.3.2.5 using 20 smoking-related and 20 affectively neutral images. Five additional smoking pictures selected for higher reactivity values were presented separately, immediately before rTMS, for craving-induction. Participants were exposed to the paradigm at baseline and post-rTMS. Cue presentation followed a fixed order of 4 blocks (smoking-neutral-smoking-neutral), each with five unique pictures presented at random. All blocks of a same cue type were paired for normative reactivity values.
Novick et al. (2022) (82)	Image	Vision (*in vitro* cues)	Participants viewed grayscale images of smoking and neutral cues. Smoking cues were images of people smoking cigarettes, holding cigarettes, and handling smoking-related items, such as lighters. Neutral cues were images matched for visual content (e.g., a person with a pen in their mouth). To ensure participant engagement, a target stimulus (picture of an animal) was presented infrequently, and participants were instructed to respond with a button press. The task consisted of 20 smoking, 20 neutral, and four target images, with each image presented for four seconds. During the interstimulus interval, a fixation point appeared on a gray screen for a variable length of time (between 6–14 s). Midway through the task, the fixation point appeared during a 24-s rest period. Stimuli class was pseudo-randomized with no more than two images of a given image type being presented consecutively. The total task duration was 10 min and 36 s.
Robinson et al. (2022) (84)	Image	Vision (*in vitro* cues)	The Dot-Probe Task (DPT) was used to assess attention bias (AB) in the laboratory, with probes following the cigarette and neutral pictures with equal probability. On the smartphone-administered modified DPT, those in the attentional bias modification group had 100% of the probes replace neutral pictures, with the intention to reduce participants’ AB to smoking cues, while those in the sham group had 50% of the probes replace neutral pictures and 50% of the probes replace smoking pictures, to avoid influencing AB.
Yang et al. (2022) (83)	Augmented reality (AR)	Vision (*in vitro* cues)	The experimental AR cues consisted of six AR smoking cues (i.e., smoking paraphernalia: cigarette, pack of cigarettes, pack and lighter, pack and ashtray, cigarette and lighter, and lit cigarette in an ashtray with smoke motion) and six AR neutral cues i.e., pen, notebook, pencil and eraser, pencil with notepad, sticky notes and pen, and soda bottle with motion of effervescence and condensation Each cue was presented for 60 s. In both conditions, a pretest AR neutral cue (i.e., pencil) was presented in the first trial to establish a baseline urge, and then a pretest AR smoking cue (i.e., cigarette) was presented in the second trial to assess pretest CR. For trials 3–26, participants in the extinction condition viewed smoking cues, whereas those in the control condition viewed neutral cues. Each set of cues (6 smoking or neutral cues) was presented four times (i.e., four blocks of six cues) in four quasi-random orders. Finally, participants in both conditions saw the posttest AR cigarette cue in trial 27, and the posttest AR pencil cue in trial 28.
Barnabe et al. (2023) (96)	Cigarette, image, video	Behavior and vision (*in vivo*/vitro cues)	Four conditions (phase 1): stress task and smoking cue, stress task and neutral cue, non-stressful task and smoking cue, or non-stressful task and neutral cue. Physiological and craving measures were collected and followed by a 10-min break. All participants then went through the extinction protocol (phase 2) which entailed four rotations of: a five-minute video with smoking-related content (composed of similar but non-identical clips to those presented in the baseline visit), a five-minute presentation of smoking images (with each image presented for 3 s, see Supplementary methods), and five minutes of manipulating smoking paraphernalia (e.g., lighter, cigarettes).

Table 6 gives a description of the smoking cue-reactivity paradigms and their types, as well as stimulus materials in these trials. In terms of types, the cue-reactivity paradigms fall into two main categories: one is the behaviorally induced craving paradigm (containing manipulative behaviors that combine visual and or olfactory sensations or purely imaginative behaviors). Manipulative behaviors are basically that participants were required to watch and smell the lighting of a cigarette (one of their favorite brands) that was placed, and then they were asked to hold the cigarette between their fingers but were not allowed to smoke it and were next instructed to extinguish it. The other category is the visually induced craving paradigm (containing physical objects, pictures, videos, virtual reality and augmented reality). For example, the picture paradigm was basically showing the subjects smoking-related pictures and neutral pictures in a certain way. Based on the results of the 30 included papers, it was found that smoking cues induced greater craving than neutral cues, both behaviorally and visually induced.

## Discussion

The review above summarizes a series of RCTs of CR in tobacco cessation therapy and focuses on a thematic overview of the types of cue-reactivity paradigms used in the trials, with the aim of assessing the effects of various cue-targeted tobacco cessation programs and summarizing the types of cue-reactivity paradigms used to date. Hence, we chose a scoping review to summarize the existing results and exploit the gaps in the current literature.

Overall, these results revealed that non-invasive brain stimulation (6 of 8 related articles) and exercise therapy (5 of 6 related articles) showed a trend of greater possibility in reducing subjective craving, when compared to the remaining therapies (11 of 26 pharmacotherapy-related articles, 2 of 11 psychotherapy-related articles, 4 of 11 other therapies related articles), regardless of variations in the number of studies conducted in each category. But due to more significant heterogeneity of studies across samples, sociodemographic information (gender, age, region), types of cue-reactivity paradigms, outcome measures and other dimensions made comparisons of the efficacy of different interventions, even the same intervention across studies, not sufficiently comparable. Even more identifiable, the measures used to assess subjective craving vary widely across studies, such as the use of the QSU-Brief, CWQ, or various types of VAS (see Tables 1–5), which further make craving in such trials challenging to measure objectively and quantitatively. The above-mentioned heterogeneity of the experimental design and implementation stage makes it challenging to compare the effect of different types of tobacco cessation interventions, further forming the situation of a lack of repetitive research. As a result, the corresponding literature only focused on the development of abstinence methods rather than the exploration of the effects. At the same time, the physiological and brain function indicators accounted for a small proportion of the reviewed articles. The physiological indicators did not show statistically significant differences in more trials. In contrast, studies based on brain function as a measure EEG and fMRI show a quantitative imbalance while their results had their own similarities and differences with non-RCTs.

Pharmacologically, the therapeutic targets under development are the endogenous cannabinoid system, nicotinic acetylcholine α4β2 and α7 subtypes, CB1 receptor neutral antagonists, fatty acid amide hydrolase inhibitors (110) and metabotropic glutamate receptor 5 (111). For example, drugs targeting the endogenous cannabinoid system have been more studied in animal experiments and less in human experiments, currently mainly cannabidiol (112). Although blocking the α4β2, but not α7 subtype has been shown to be effective in reducing nicotine intake in animal studies, blocking the α7, but not α4β2 isoform of the nicotinic acetylcholine receptors reversed cue-triggered nicotine relapse behavior (113). Current studies have developed tobacco cessation medications in addition to those summarized in the results section, such as naloxone (114) which has mostly been found to reduce craving. Franklin et al. (45) found that varenicline diminished smoking cue-elicited ventral striatum and mOFC responses, and Ketcherside et al. (8) found that baclofen mitigates the reward response to smoking cues through an increase in tonic activation of the DLPFC, an executive control region, and the aforementioned altered neural activity correlated with cue-induced craving. However, no clear findings have been made on the pathways by which drugs mediate different manifestations of cue-induced craving, and more drugs with different chemical structures need to be developed. Previous studies need to be repeated to explore the associated addictive mechanisms and ensure the safety of drug treatments and their effectiveness.

Non-invasive brain stimulation was primarily tDCS and rTMS, with rTMS being one of the most effective methods found to reduce cigarette smoking in the intervention group, but neither technique significantly improved outcomes of tobacco cessation rate (115). Based on fMRIs, rTMS (109) and tDCS (116, 117) targeting the DLPFC were found to be the most effective in reducing cravings by reducing activity in the right insula and right thalamus as well as reducing rsFC between the left DLPFC and the mOFC for rTMS. Zangen et al. (9) found rTMS bilaterally stimulating neural pathways in the lateral prefrontal cortex and insula with an intensity above the neuronal threshold for activation can also reduce cigarette craving. Therefore, non-invasive brain stimulation has multiple targets for reducing cue-induced cravings. Further exploration of the mechanism of non-invasive brain stimulation in the treatment of TUD will provide a better basis for improving the reliability and efficiency of treatments.

In psychotherapy, there are mainly mindfulness (118), hypnosis-based treatment (119), cognitive behavioral treatment (120), cue exposure treatment (CET) (81) and psychological paradigm training, which are mainly neurofeedback training (7, 106, 121), retrieval-extinction (67) and ABM (103). These psychotherapies are mainly used to achieve tobacco cessation, or relapse prevention, by reducing smoking cue-induced craving and or the impulsivity to smoke. Although the 6 psychotherapeutic articles included in this scoping review did not find a reduction in craving or modulation of cognitive biases, this does not mean that various psychotherapies are not effective in this regard, when there may be related to individual subjective perception thresholds and different matches with different psychotherapies. On the other hand, Kim et al. (106) and Froeliger et al. (53) found corresponding psychotherapy activity changed in relevant brain regions under fMRI scan, while Andreu et al. (105) found that psychotherapy exhibited different effects on different components of ERP. In summary, psychotherapy can further help to improve substance use disorder (SUD) symptoms and prevent relapse by regulating brain function. This requires future research to strengthen the mechanism of SUD psychotherapy, from brain function and pathophysiological indicators, in order to develop higher physiological and imaging indicators with higher specificity, to compensate for the shortcomings of subjective measures.

For exercise therapy and other treatments, nearly all exercise therapy and approximately half of combination therapies showed the effect of reducing subjective craving, while the other combination therapies were not found to be significantly different from controls in the reviewed literature. However, it is still an integrative treatment approach that has received more attention from researchers and is consistent with the treatment philosophy of the bio-psycho-social medical model. Mondino et al. (122) found that combining transcranial alternating current stimulation and ABM helped smokers wishing to quit smoking reduce craving, attention and impulsive decision-making to smoking cues. Otto et al. (81) found that d-cycloserine enhanced the efficacy of CET in reducing cue-induced craving. In summary, given the variations in the effects of different combinations of treatment modalities for tobacco cessation, further exploration of the interactions and similarities in the mechanisms of multimodal combinations is needed to find more comprehensive and personalized approaches to tobacco cessation.

It is worth mentioning that virtual/augmented reality related treatment is one emerging form of smoking cessation intervention targeting cue-reactivity. To our knowledge, most studies found that virtual/augmented reality related smoking cue-paradigms can provoke cue-reactivity, especially craving (25, 30, 123–126). And the technology of virtual/augmented reality is mainly applied to CET (83, 108, 127). However, many studies, especially virtual reality related studies, aimed at assessing the effects of virtual/augmented reality CET on smoking-related cue-reactivity were quasi-experimental studies without using a control group (128–131), or the RCT study did not report the results of cue-reactivity between groups (132), and most of them found that virtual/augmented reality CET could reduce craving. Notably, the two included articles (83, 108) in our review had no significant difference between groups in craving. Overall, the effect of virtual reality (VR) CET in craving is mixed, which is also reported in a systematic review (127), while there are not enough augmented reality (AR) CET studies to make a similar conclusion. So, the potential of VR-or AR-based smoking cessation intervention is needed to be fully explored.

The cue-reactivity paradigms as the primary means of eliciting smoking craving in experiments shows significant variability in the reviewed articles, reducing the cross-sectional comparability of the effects of various tobacco cessation treatment experiments. The materials used by researchers to stimulate smoking cravings were homemade (7) or modified from other researchers’ galleries (35), from tobacco ads^1^ (114), queried from google images for ‘positive smoking’ and ‘negative smoking’ (133) or other sources such as the Normative Appetitive Picture System (NAPS) (134) or the International Smoking Image Series (ISIS) (135). Most home-grown stimulated smoking craving images are used for their own experiments, making it difficult to conduct replicated studies. To address these challenges, researchers such as Manoliu (135) generated and validated a large set of individually rated SRC to assess different dimensions of stimulus intensity, including craving, valence and arousal. Thus, they proposed a novel image bank that rates the three dimensions of craving, valence and arousal on a continuous scale, which not only provides a good description of a publicly available rating software but contributes to the scientific field.^2^ There are only 250 images in the image library, but there are many types of smoking cue materials used in the study, such as pictures, videos, audio, physical cigarettes, virtual or augmented reality simulations of cigarette tools or smoking scenes (25). In addition, the materials used as controls for the study also vary, such as neutral materials, negative emotion materials, positive emotion materials, food materials, stress materials, and aversion materials. Therefore, it is better to expand the smoking and controlled cue material library. Besides that, due to cultural and individual differences, the need for a uniform and standardized database of smoking cue materials has become imperative.

## Limitations

To begin with, the selection of included RCTs and the use of strict inclusion criteria to ensure the relative quality of the review is inevitably biased by the lack of quality control of the included pieces of literature. In addition, the exclusion of literature on TUD with co-morbidities prevents us from demonstrating how CR is affected in the context of comorbidities. However, numerous studies (136–140) suggest that the prevalence of TUD is higher in individuals with associated psychological problems or psychiatric disorders. Most studies (5, 141–144) on the relationship between TUD co-morbidity and CR have shown that individuals with TUD with comorbidity have difficulties quitting and that co-morbidity objectively alters the performance of CR. Therefore, to make tobacco cessation treatment more personalized and comprehensive, comorbidity research should be strengthened to deconstruct the mechanism of regulating brain addiction of TUD with comorbidities, which will be a challenging study. Furthermore, our literature search strategy and limited database selection may have resulted in the omission of literature that met the inclusion criteria, thus preventing this review from providing a comprehensive overview of current advances in smoking cessation therapy based on CR. And we only searched for publications in English and Chinese, which led to missing literature in other languages and further contributed to the abovementioned problems. Finally, there is also a limitation with regards to the differences among the included articles in gender/sex ratio, ethnicity or region or origin or diagnostic criteria of the study participants, sample size of the individual studies, as well as the statistical methods, resulting in significant heterogeneity among various studies. Therefore, we did not statistically test for the overall efficacy, which is also a limitation for a descriptive and comparative approach we adopted here.

## Conclusion

This paper reviews the effects of various cue-reactivity-targeted smoking cessation therapies and types of cue-reactivity paradigms to understand the role of cue-reactivity in smoking cessation diagnosis and treatment. It proposes that, given that current studies are still inadequate in terms of homogeneity and lack repeated validation, cue-reactivity can be conducted in the future by constructing a standard library of smoking cue materials and conducting cue-reactivity causal analysis in order to adequately screen for causes of addiction persistence. In summary, the following problems remain: (1) it is still challenging to find specific targets among the factors influencing cue-reactivity, and it cannot be ruled out that they are due to a combination of factors, so causality studies need to be strengthened; (2) the specificity of the indicators can be enhanced by expanding the sample size, strengthening the homogeneity of the sample, standardizing the parameters of the cue-reactivity paradigms, increasing the years of follow-up, and standardizing statistical methods; (3) there is a lack of a unified and standardized database of smoking cues worldwide, and the construction of a database of smoking cues would be a worthwhile endeavor to facilitate repeat trials and the reliability of final scientific findings. Data-driven approaches toward addiction have been increasing in recent years, which could allow for the personalization of big data analysis and the differentiation of responses, such as craving levels between different paradigms, providing practical technical support for the search for a more stable and effective cue-reactivity paradigms.

## Data availability statement

The original contributions presented in the study are included in the article/Supplementary material, further inquiries can be directed to the corresponding author.

## Author contributions

ML: Conceptualization; data curation; investigation methodology; project administration; visualization. QG: Conceptualization; supervision; validation. YF: Funding acquisition; supervision. ZC: Conceptualization; funding acquisition; supervision. All authors contributed to the article and approved the submitted version.

## Funding

This study was supported by the National Natural Science Foundation of China (NSFC) (Nos.32060196, 82201597, 31760281, and 81760258) and Yunnan Ten Thousand Talents Plan Young and Elite Talents Project (YNWR-QNBJ-2018-027).

## Conflict of interest

The authors declare that the research was conducted in the absence of any commercial or financial relationships that could be construed as a potential conflict of interest.

## Publisher’s note

All claims expressed in this article are solely those of the authors and do not necessarily represent those of their affiliated organizations, or those of the publisher, the editors and the reviewers. Any product that may be evaluated in this article, or claim that may be made by its manufacturer, is not guaranteed or endorsed by the publisher.

## Footnotes

^1^ http://www.gauloises.com/

^2^The image database and their ratings are available at https://smocuda.github.io/.
