# Supplementary material for: Cue-reactivity targeted smoking cessation intervention in individuals with tobacco use disorder: a scoping review
Source: Front Psychiatry. 2023 Sep 7;14:1167283. doi: 10.3389/fpsyt.2023.1167283 (PMC10512743; doi:10.3389/fpsyt.2023.1167283)
Supplement: Supplementary file 1 [file Table_1.DOCX]

**Table S1.** Data sources and search strategy

| **Databases** | **Query** |
| --- | --- |
| PubMed | ("cue"[Text Word] AND ("nicotine"[Text Word] OR "tobacco"[Text Word])) AND (humans[Filter]) |
| Embase | ('cue'/exp OR 'cue') AND ('nicotine'/exp OR 'nicotine' OR 'tobacco'/exp OR 'tobacco') AND [humans]/lim |
| CNKI | (篇关摘: 烟草成瘾 (精确) ) OR (篇关摘: 烟瘾 (精确) ) OR 篇关摘: 吸烟 (精确) ) AND 篇关摘: 线索(精确) ) |
| Wanfang | 主题:(烟草成瘾or烟瘾or吸烟) and主题:(线索) |

Note: We searched these four databases until April 2023.

**Table S2.** The content of diagnosis criteria as well as excluded criteria of medication and comorbidities added to the Tables 1-5.

|  | | **Diagnosis criteria (smoking status)** | **Excluded medication** | **Excluded comorbidities** |
| --- | --- | --- | --- | --- |
| Tiffany et al. (2000) [70] | | NG (smoking at least a pack of cigarettes per day) | Currently using any medication | Any conditions that would contraindicate the use of a nicotine transdermal patch |
| Shiffman et al. (2003) [71] | | NG (light smokers smoking 11-24 cigarettes per day and heavy smokers smoking more than 24 cigarettes per day) | NG | A history of or current allergy to adhesives; severe skin disease, or frequent skin rashes, had abused drugs or alcohol within the last 3 months; had a history of severe mental illness; had lung cancer or unstable cardiovascular disease (e.g., unstable angina, severe congestive heart failure, or uncontrolled hypertension); or had used supplemental oxygen |
| Hutchison et al. (2004) [57] | | NG (average no. cigarettes/day: placebo group: 15.2±7.0, olanzapine group: 15.6±4.5) | NG | Had a breath alcohol level of zero before each session, no contraindications for the use of the study medications |
| Waters et al. (2004) [72] | | NG (smoking for at least 5 years and  to be smoking at least 15 cigarettes per day) | NG | NG |
| Mahler et al. (2005) [58] | | NG (smoking an average of 21±5.4 cigarettes/day, had been smoking for an average of 12±7.6 years) | NG | NG |
| Morissette et al. (2005) [73] | | NG (daily moderate to heavy smokers who smoking 15-40 cigarettes per day) | A previous history of using the nicotine patch; currently taking anxiolytics, antidepressants, cardiac medications (e.g., beta blockers), asthma medication, or over-the-counter diet medications | Endorsed a medical history that contraindicated use of the nicotine patch (e.g., heart disease, high blood pressure, allergy to adhesive tape, pregnancy); possible alcohol or substance disorders by scoring 6 or higher on the Drug Abuse Screening Test or greater than 12 on the Alcohol Dependence Scale; endorsed current Axis I disorders |
| Niaura et al. (2005) [74] | | NG (smoking 10-24 cigarettes a day continuously for the last 3 years) | Use of narcotics or anxiolytics within 30 days of study enrollment; introduction of antidepressants in the last three months; use of St John’s Wort or kava-kava within 3 months; use of xanthine-derived bronchodilators, sympathomimetic agents, or alpha-adrenergic blocking agents; prescription medication for asthma (excluding inhaled steroids and inhaled beta agonists); had used any investigational drug within 30 days; and medication which, in the opinion of the investigators, could interfere with the conduct of the study | Routine use of tobacco other than cigarettes; use or consumption of tobacco, alcohol or caffeine/food within 10, 24 or 1 hour(s), respectively, before the test session; alcohol or drug abuse within 3 months of study enrollment; medical history of congestive heart failure, angina or an acute cardiovascular disorder; chronically dry mouth; active esophagitis or peptic ulcer disease; uncontrolled hyperthyroidism or insulin-dependent diabetes mellitus; history of severe mental illness; and medical condition which, in the opinion of the investigators, could interfere with the conduct of the study |
| Reid et al. (2007) [59] | | NG (smoking at least 15 cigarettes/day) | Enrollment in smoking cessation treatment | Evidence of current depression (BDI >17), current drug or alcohol dependence, current axis-I psychiatric disorders which require medication treatment, and current, or within the last 6 months |
| Liu et al. (2009) [97] | | NG (smoking 5-10 cigarettes/day and scoring <5 on the FTND for light smokers; or smoking ≥15 cigarettes/day continuously for at least 12 months prior to screening and scoring ≥6 on the FTND for heavy smokers) | Current use of antidepressants; self report of treatment with any prescription drug during the previous 2 weeks or with any over-the-counter drug during the 3 days prior to the experimental session | Abuse of or dependence on other drugs (opioid and methamphetamine); current or past history of DSM-IV Axis I disorders; current diagnosis of depression; clinically evident cognitive impairment |
| Hussain et al. (2010) [91] | | NG (smoking ≥10 cigarettes/day) | regular users of psychotropic medications | Had psychiatric comorbidity, current substance abuse or dependence |
| Brandon et al. (2011) [60] | | NG (smoking at least 15 cigarettes daily) | Using other smoking cessation medications | Current mood or psychotic disorders |
| Culbertson et al. (2011) [61] | | DSM-IV (smoking ≥10 cigarettes per day) | Current use of medications that could alter brain function | History of any Axis I psychiatric diagnosis other than nicotine dependence, medical conditions that might affect brain function, current illicit drug use other than occasional use of marijuana |
| Franklin et al. (2011) [45] | | NG (“nicotine-dependent smokers”; pack years:  12.6±2.1) | NG | With other current substance dependence, current Axis I DSM-IV psychiatric diagnoses, significant medical conditions, an intellectual ability estimate score of 80 or less on the Weschler Abbreviated Scale of Intelligence, an abnormal structural MRI, or a history of head trauma or injury causing loss of  consciousness lasting longer than 3 minutes or associated with skull fracture or intercranial bleeding or who had irremovable magnetically active objects on or within their body |
| Ditre et al. (2012) [62] | | DSM-IV (smoking at least 20 cigarettes per day) | Use of any pharmacological agents that might impact treatment outcome | A diagnosis of schizophrenia, bipolar disorder, current major depression, dementia, or nonalcohol drug dependence |
| Hitsman et al. (2013) [46] | | NG (smoking at least 20 cigarettes/day on average during the prior year) | Treatment with another investigational drug within 1 month of enrollment or plans to take another investigational drug within 1 month of study completion; current treatment with antidepressant, antipsychotic, or antianxiety medication; use of nicotine replacement products or bupropion within the last 3 months | History of alcohol or drug abuse/dependence (other than tobacco); history of seizures, including febrile seizures, childhood seizures, and seizures associated with alcohol withdrawal; and any history of clinically significant medical or psychiatric disease |
| Du et al. (2014) [77] | | NG (low dependence smokers who smoked their first cigarette more than 30 min after waking up, and smoked on a daily basis for at least 1 year.) | Had been treated with known enzyme altering agents or any NRT or any other treatment for smoking cessation within 30 days of the craving provocation visit; had used any prescription psychiatric medications within 14 days of the craving provocation visit; had used any medication that affects the central nervous system, such as sedating antihistamines, within 24 h of the craving provocation visit; had used any nicotine containing products at any time during the  provocation/treatment visit; and had used any product which could jeopardize the safety of the participant or impact on the validity of the study results | Had known or suspected intolerance or hypersensitivity to nicotine or any other ingredients in formulation; had current or recent history (within the last 1 year) of alcohol or other substance abuse; had any disease that might interfere with the absorption, metabolism or excretion of nicotine |
| Rabinovitz et al. (2014) [102] | | DSM-IV (smoking >10 cigarettes per day for the past 12 months) | Used chronic medications or food supplements in the past three months | Any DSM IV Axis I disorders other than nicotine dependence |
| Schlagintweit et al. (2014) [94] | | NG (dependent smokers FTND ≥3, smoking an average of 14 cigarettes per day) | Used NRTs at the time of participation or had prior experience with oral NRTs (the gum or lozenge) | Past or present mental illness including substance use disorders (excluding nicotine dependence) |
| Pachas et al. (2015) [47] | | DSM-IV (smoking 10 or more cigarettes per day for the prior 3 months) | a medical contraindication to propranolol administration or concurrent use of β-blockers, antiarrhythmic agents, calcium channel blockers, varenicline, bupropion or nicotine replacement therapy | Had systolic blood pressure <100 mm Hg at screening; met DSM-IV criteria for a substance use disorder other than nicotine or caffeine or had evidence for recent use of addictive drugs other than nicotine or caffeine; had a lifetime history of psychotic or bipolar disorder; or met criteria for major depressive disorder or posttraumatic stress disorder in the prior 6 months |
| Haarmann et al. (2016) [107] | | NG (displaying at least moderate nicotine dependence, FTND ≥5) | Taking drugs impacting on sympathovagal balance (e.g., beta blockers, selective serotonin reuptake inhibitors, theophylline) | Suffering from diseases known to increase sympathetic activity (e.g., heart failure, COPD, depression, obstructive sleep apnoea, pulmonary-arterial hypertension) |
| Miller et al. (2016) [63] | | NG (smoking an average of 6 cigarettes per day, nicotine dependence assessed using FTND) | NG | Current or past year diagnosis of a DSM-IV Axis I disorder (assessed using the non-patient version of the Structured Clinical Interview for DSM-IV), excluding nicotine dependence; high blood pressure or a history of cardiovascular problems |
| Gendy et al. (2018) [92] | | NG (smoking at least 10 cigarettes per day for at least 2 years) | Had received treatment for nicotine dependence in the past 3 months, use of psychoactive drugs as revealed by urine toxicology | A history of drug or alcohol dependence within the last 5 years, consumption of more than 15 alcoholic drinks per week on average during the past month use of any illicit drug more than once per week on average during the past month, any history of or current cardiovascular, hepatic or renal disease, diabetes |
| Nides et al. (2018) [56] | **Study 1** | NG (smoking at least 5 cigarettes per day, light/low-dependency smokers who smoked ≤20 cigarettes per day, which is the population for which the 1.5-mg nicotine mini lozenge is indicated, and heavy/high-dependency smokers who smoked >20 cigarettes per day, which is the population for which the 4-mg nicotine mini  lozenge is indicated) | Treated within 30 days with known liver enzyme-altering agents, who used any prescripion psychoactive medication within 14 days, took a sedating over-the-counter medication within 24 hours, or currently used NRT | Had a history of alcohol or drug abuse within the previous year, |
|  | **Study 2** | NG (smoking >20 cigarettes per day for at least one year) | The same as **Study 1** | The same as **Study 1** |
| Versace et al. (2019) [48] | | NG (smoking at least five cigarettes per day) | Taking psychotropic medication | Having a lifetime history of a psychotic disorder, being hospitalized in the past year for a psychiatric condition, being involved in a concurrent smoking cessation program, having a cur rent psychiatric disorder, scoring high or moderate on a suicidality scale of the Mini-International Neuropsychiatric Interview |
| Ketcherside et al. (2020) [8] | | NG (smoking at least 10 cigarettes per day for the past 6 months) | Use of any other smoking cessation strategies | Any current severe psychiatric symptoms, or diagnosis of any substance use disorders (SUDs) other than nicotine, current Axis 1 DSM-IV disorder, an intellectual ability estimate score below 80 |
| Kotlyar et al. (2020) [80] | | NG (smoking at least 8 cigarettes on an average day) | Use of any medication that could interfere with study measures (e.g., psychoactive medications), use of smoking cessation therapy during the month previous to screening | Current unstable medical or psychiatric conditions; a history of severe motion sickness |
| Lawson et al. (2021) [49] | | NG (smoking at least 10 cigarettes per day for the past 6 months) | Use of smoking cessation medication, including nicotine replacement therapy, in the past 14 days; medical treatment for substance use in past 3 months, including Suboxone (buprenorphine) and methadone (at phone screen) | Use of other tobacco products, including e-cigarettes, in past 7 days; substance use:  alcohol, using a combination of the National Institute on Drug Abuse (NIDA) modified ASSIST (4-26 = moderate risk; 27+ = high risk) and urine toxicology screen (both at intake): Cannabis,  Methamphetamine, Prescription stimulants, Opioids |
| Novick et al. (2022) [82] | | NG (FTND ≥3, smoking at least 10 cigarettes per day over the past year) | Current regular use of any tobacco products other than cigarettes; current regular use of psychotropic medication | Current psychiatric or major medical illnesses; lifetime history of psychotic disorder, other Axis 1 psychiatric disorder within past year, lifetime history of substance dependence disorder other than nicotine, history of substance use disorder other than nicotine within past 2 years (DSM-IV); heavy alcohol use within past year (>7 drinks/week or >3 drinks/occasion for females; >14 drinks/week or >4 drinks/occasion for males); contraindications to progesterone use (e.g., thrombophlebitis, stroke); metallic implants or claustrophobic (contraindication for fMRI) |
| **Table 2 authors (year)** | | **Diagnosis criteria (smoking status)** | **Excluded medication** | **Excluded comorbidities** |
| Fregni et al. (2008) [100] | | NG (currently smoking 15 or more cigarettes per day for at least 1 year) | Were taking any psychiatric medication | Had any neuropsychiatric disorder or current or past history of alcohol or other drug abuse |
| Boggio et al. (2009) [50] | | NG (currently smoking ≥10 cigarettes per day for at least 1 year) | Were taking any  psychiatric medication | Had any neuropsychiatric disorder, current or past history of alcohol or other drugs abuse |
| Li et al. (2013) [64] | | NG (smoking ≥10 cigarettes/day) | Current use of nicotine replacement therapy, bupropion, or varenicline; taking medications that lower the seizure threshold | Use of other tobacco products; medical conditions; nonnicotine substance dependence or abuse; a history of psychiatric disorder; with any implanted metal devices (e.g., pacemakers, metal plates, wires) |
| Meng et al. (2014) [98] | | NG (at least two years of smoking history with more than 8 cigarettes daily) | Currently not on medication with active central nervous system properties | History of major neurological disorders (e.g., seizure, dementia,  Parkinson’s disease, depression); brain injury or stroke history; vision diseases; chronic pain or hyperalgesia |
| Li et al. (2017) [115] | | DSM-IV-TR (smoked ≥10 cigarettes/day) | Currently used nicotine replacement therapy, took smoking cessation medications, took any other psychoactive medications | Used tobacco products other than cigarettes; had any unstable medical conditions, had current or past DSM-IV-TR Axis I disorders |
| Yang et al. (2017) [99] | | NG (FTND ≥4; smoking at least 10 cigarettes per day in the past two years) | Received pharmacotherapy in the past two months | History of major neurological disorders (e.g., seizure, dementia, Parkinson’s disease, depression); brain injury or stroke history; vision diseases; chronic pain or hyperalgesia |
| Li et al. (2020) [65] | | NG (smoking ≥10 cigarettes per day, nicotine dependence as determined by the FTND) | Other forms of nicotine delivery, such as nicotine patch, electronic cigarettes | Current dependence, defined by DSM-V criteria, on any psychoactive substances other than nicotine or caffeine; history of neurological disorder or seizure, increased intracranial pressure, brain surgery, or head trauma with loss of consciousness for >15 minutes, implanted electronic device, metal in the head; history of autoimmune, endocrine, viral, or vascular disorder affecting the brain; History or MRI evidence of neurological disorder that would lead to local or diffuse brain lesions or significant physical impairment; unstable cardiac disease, uncontrolled hypertension, severe renal or liver insufficiency, or sleep apnea; life time history of major Axis I disorders such as: Bipolar Affective disorder, Schizophrenia, Post-traumatic Stress disorder or Dementia or Major Depression; self report of >21 standard alcohol drinks per week in any week in the 30 days prior to screening |
| Zangen et al. (2021) [9] | | DSM-5 (at least 10 cigarettes/day for at least 1 year) | Current treatment for smoking, use of any psychotropic medication on a regular basis | Use of nicotine other than through cigarettes, any other active psychiatric disorder diagnosed according to the DSM-5, any other substance use disorder during the last 12 months before recruitment, history of epilepsy or seizures (except those therapeutically induced by electroconvulsive therapy) or increased risk of seizures for any reason, any significant neurological disorder or insult, history of any metal in the head (outside the mouth) or metallic implant |
| Marques et al. (2022) [101] | | NG (FTND >6) | Present use of bupropione, varenicline or nortriptyline | Other substance use disorders, moderate or severe psychiatric comorbidity on Patient Health Questionnaire-9, Generalized Anxiety Disorder 7-item or clinical interview, any reported neurological disorders |
| **Table 3 authors (year)** | | **Diagnosis criteria (smoking status)** | **Excluded medication** | **Excluded comorbidities** |
| Bowen et al. (2009) [52] | | NG (Cigarettes smoked per day: control group: 4.73±4.40, intervention group: 5.93±4.79 ) | NG | NG |
| Kim et al. (2015) [106] | | NG (>5 years of smoking, smoking >10 cigarettes per day, FTND >4) | The use of tobacco products other than cigarettes (e.g., nicotine patches/gum) and current use of a nicotine replacement therapy, such as bupropion, varenicline, or nortriptyline | NG |
| Elfeddali et al. (2016) [103] | | NG (smoking on a daily basis for at least 1 year) | NG | Did not understand (ability to read and write) Dutch well, when they consumed more than 16 standard units of alcohol per week (10 g of pure alcohol in the Netherlands), when they were unable to refrain from alcohol use during a whole day, when they regularly used drugs or calming medicines, when they reported having a psychological disorder or dyslexia and when they were color blind and/or vision impaired (even with glasses) |
| Hartwell et al. (2016) [66] | | The Minnesota International Neuropsychiatric Interview (nicotine-dependent smokers, ≥10 cigarettes/d) | Use of other tobacco products; current use of nicotine replacement therapy, bupropion or varenicline; medications that could affect brain function | Medical conditions that could affect brain function; current or past DSM-IV Axis I disorders |
| Froeliger et al. (2017) [53] | | NG (nicotine-dependent adult smokers, smoking > 10 cigarettes/day for a minimum of 2 years) | Current use of prescription medications that affect the central nervous system (e.g., blood pressure medication) or BOLD response | A past head injury or primary neurological disorder associated with MRI abnormalities; physical or intellectual disability affecting completion of assessments; use of illicit substances or abuse of prescription medications within the last month; current or past psychosis; blood alcohol level (BAL) of more than 0.0 on more than one occasion |
| Germeroth et al. (2017) [67] | | DSM-IV (smoking 10 or more cigarettes per day for 3 years or more) | Taking beta-blockers, anti-arrhythmic agents, psychostimulants, or any other agents known to interfere with heart rate or blood pressure | Substance dependence other than nicotine in the past 60 days; current/active (untreated) psychotic disorder, current major depressive disorder, bipolar affective disorder, or a severe anxiety disorder assessed with the Mini International Neuropsychiatric Interview |
| Andreu et al. (2018) [105] | | NG (smoking daily) | NG | Current abuse of a substance other than nicotine, a current diagnosis of a physical or psychiatric illness |
| Bu et al. (2019) [7] | | NG (smoking 10 or more cigarettes per day for 2 years or more) | Treatment with any drugs during the previous 3 months | Chronic neurological, psychiatric, or medical conditions |
| Malbos et al. (2022) [108] | | DSM-5 (with the presence of at least three of the 11 DSM-5 criteria for nicotine dependence) | Simultaneous use of any concurrent method of tobacco abstinence (i.e. NRT, smoking cessation drugs such as bupropion or varenicline, electronic cigarettes) | Unstable physical or psychiatric disease and contraindications to virtual reality therapy such as photosensitive epilepsy |
| Yang et al. (2022) [83] | | NG (currently smoking ≥ 3 cigarettes per day for the past year) | NG | Regular use of other tobacco products (>30% of the time) |
| Barnabe et al. (2023) [96] | | NG (scoring 5 or higher on the Fagerström Test for Cigarette Dependence) | Current treatment with ß-blockers or psychostimulants | Moderate to severe psychiatric disorders: lifetime history of psychoses or current post-traumatic stress disorder, mood disorder, panic or other anxiety disorder |
| **Table 4 authors (year)** | | **Diagnosis criteria (smoking status)** | **Excluded medication** | **Excluded comorbidities** |
| Taylor et al. (2007) [85] | | NG (had smoked at least 10 cigarettes daily for the past 3 years) | NG | NG |
| Janse Van Rensburg et al. (2009a) [86] | | NG (smoked at least 10 cigarettes a day, had been a regular smoker for 2 or more years) | NG | Had injury or illness that would inhibit their ability to safely exercise at a moderate intensity |
| Janse Van Rensburg et al. (2009b) [87] | | The same as Janse Van Rensburg et al. (2009a) | NG | The same as Janse Van Rensburg et al. (2009a) |
| Elibero et al. (2011) [68] | | NG (smoking at least 10 cigarettes/day for at the least the past year) | Taking benzo diazepines, antidepressants, neuroleptics, stimulants, anticon vulsants, or beta agonists | Contradictions for moderate-intensity exercise based on the Physical Activity Readiness Questionnaire |
| Janse Van Rensburg et al. (2013) [54] | | NG (smoking 10 or more cigarettes a day for the past 2 years) | NG | Had any illness or injury that would inhibit them from exercising safely |
| Fong et al. (2014) [93] | | NG (smoking an average of 10 cigarettes or more per day for at least two years) | NG | Had no contraindications to physical activity as determined by the Physical Activity Readiness Questionnaire |
| **Table 5 authors (year)** | | **Diagnosis criteria (smoking status)** | **Excluded medication** | **Excluded comorbidities** |
| Hutchison et al. (1999) [69] | | NG (smoking at least 20 cigarettes per day) | Currently taking medications with opiates | A history of opioid dependence, were positive on a urine opiate screen, had liver function tests (SGOT, bilirubin) greater than three times normal, had any symptoms of an acute medical problem, or had a chronic medical problem that could contraindicate participation (e.g., cardiac disease) |
| Sayette et al. (1999) [55] | | NG (an average 18.4 cigarettes per day, had smoked for an average of 5.6 years) | NG | NG |
| Rohsenow et al. (2007) [75] | | NG (smoking at least 15 cigarettes per day for at least 6 months) | In a quit attempt or using nicotine replacement; use of medications that contraindicated opiate antagonists; use of medications that would interfere with reactivity assessment (including bupropion, anticholinergics, antihistamines, tricyclic antidepressants, anxiolytics, hypnotics, beta blockers or agonists, or alpha blockers) | A history of opioid abuse or dependence; liver function tests greater than three times normal; a medical condition that precluded participation; allergy to adhesives or acetaminophen |
| Santa Ana et al. (2009) [76] | | NG (smoking ≥10 cigarettes/day for the past 12 months) | Use of psychoactive medication, or medications for smoking cessation, such as nicotine replacement, bupropion, or varenicline | Psychiatric or medical illness, serious neurological or seizure disorder, use of psychoactive drug or alcohol dependence, known allergy to D-cycloserine |
| Kamboj et al. (2012) [88] | | NG (smoking at least 15 cigarettes per day, FTND ≥4) | NG | With a history of substance dependence or medical or psychiatric difficulties or epilepsy, renal failure or porphyria |
| Havermans et al. (2014) [104] | | NG (smoking ≥10 cigarettes a day for at least 1 year) | Use of psychotropic medication | History of physical or mental illness; history of drug or alcohol abuse |
| Begh et al. (2015) [90] | | NG (smoking at least 10 cigarettes per day) | Are currently using nicotine replacement therapy (NRT), bupropion, nortriptyline, mecamylamine, reserpine, or varenicline, or undergoing any  treatment for tobacco dependence (e.g. acupuncture) that they are not willing to cease using and instead use study medication; | A medical condition that prevents them from seeing the computerised images properly, attending to the task, or pressing the keyboard buttons on the computer accurately, or completing any other study procedures; have previously had severe skin reactions to nicotine patches or severe eczema or other skin diseases that make patch use hazardous or undesirable; having a severe acute or chronic medical or psychiatric condition or previously diagnosed clinically important renal or hepatic disease |
| Das et al. (2015) [89] | | NG (FTND ≥4, smoking >10 cigarettes every day) | NG | Current/history of mental health or neurological conditions, concurrent addiction to any other substance; use of any illicit drug more than once per week, use of ketamine more than once per month; compromised renal or hepatic function |
| Schlagintweit et al. (2016) [95] | | NG (smoking an average of 12.75 cigarettes per day and had been daily smokers for at least one year) | NG | NG |
| Jones et al. (2017) [78] | | DSM-IV (smoking 15 or more cigarettes per day) | NG | Physically dependent on any drugs other than nicotine or caffeine. |
| Brandon et al. (2018) [79] | | NG (smoking ≥10 cigarettes/day for the past year) | Previous use of varenicline; use of other smoking cessation medications within the past 3 months | Renal dysfunction; history of seizures; medically at risk in the judgment of the study physician; current psychiatric disorder, including depression, bipolar, psychotic disorders, or substance/alcohol use disorder |
| Otto et al. (2019) [81] | | NG (smoking at least 10 cigarettes per day for the prior 6 months) | Use of isoniazid or ethionamide | current unstable medical illness; major depressive episode or substance use disorder other than nicotine or caffeine active in the prior 6 months; a lifetime history of any other Axis I psychiatric illness |
| Robinson et al. (2022) [84] | | NG (smoking an average of five or more cigarettes or little cigarettes per day for 30 days prior to phone screen) | Currently taking psychotropic, anticonvulsive, or narcotic medication | Had a history of neurological illness or closed head injury, reported diagnosis of seizure disorder, reported uncorrected vision problems (including color blindness), tested positive on a urine drug screen for drugs of abuse/potential abuse |

Note: not given (NG); FagerstrÖm Test for Nicotine Dependence (FTND); transcranial direct current stimulation (tDCS); M±SD: mean±standard deviation; memantine (MEM); placebo (PLAC); reactivation of smoking MMMs (REACT); functional Magnetic Resonance Imaging (fMRI); real-time fMRI neurofeedback (rtfMRI-NF); blood oxygen level dependent (BOLD); Diagnostic and Statistical Manual fourth edition (DSM-IV-TR); repetitive transcranial magnetic stimulation (rTMS); Nicotine replacement therapy (NRT); Beck Depression Inventory (BDI).

**Table S3.** The categories of smoking cue reactivity paradigms.

| **Ranking** | **Types** | **A typical description** |
| --- | --- | --- |
| 1 | 31 trials based on vision (in vitro cues) | The 60 images (smoking and neutral images) were randomly presented in each scanning session and for each participant using E-prime software. Images were viewed on a screen placed at the foot of the scanner via a mirror mounted on the head coil. Each image was presented for 3 s. A button, placed in each of the participant’s hands, was pressed upon presentation of the image to ensure attentional focus. The button-press for smoking or neutral images was randomized for hand dominance between participants. After each image, a white screen with a black fixation cross was presented for a randomly determined period of 8, 10, or 12 s, and participants were asked to view this between smoking images to remain focused. The duration of image presentation and the inter-stimulus-interval chosen fall between those reported in other event-related studies in this area [86]. |
| 2 | 20 trials based on behaviour (in vivo cues) | A tray holding an inverted plastic bowl was placed on the participants’ desk. This bowl covered cigarettes, an ashtray, and a lighter. From the control room, the experimenter instructed participants to remove the cover, light the cigarette without putting it in their mouths, and stare at it for 10s. After 10s, participants verbally rated their urge to smoke on the 0-100 scale. Participants were next instructed to extinguish the cigarette [55]. |
| 3 | 8 trials based on behaviour and vision (in vivo/vitro cues) | For the cigarette manipulation cue, subjects were instructed to open a pack of their favored brand of cigarette, pick up a cigarette, place it in their mouths, pick up a lighter, and pretend to light and smoke the cigarette. These procedures were standardized to be performed in 30 seconds. Subjects were then asked to put the cigarette away and were shown a movie of 5 minutes’ duration presenting people smoking in a pleasant way. (Six different equivalent movies were randomized across subjects, as the subjects were exposed to a different movie before and after the 3 types of treatment) [100]. |
| 4 | 2 trials based on behaviour (In vitro cues) | Participants listened to and imagined each of the scripts with their eyes closed. For demonstration purposes, a practice script was first presented. Four types of experimental imagery scripts were then presented: (a) anxiety plus smoking cues, (b) anxiety cues alone, (c) smoking cues alone, and (d) neutral cues. Two scripts of each type were used, totaling eight imaginal scenarios. Scripts were counterbalanced for both order and sequence. Each script sequence consisted of a 30-s baseline period, 50-s script presentation period, and 30 s of active imagery by the participant terminated with the word “stop.” Participants were then asked to open their eyes and complete postexposure trial questionnaires asking them about how they felt during the most recent scenario [73]. |
| 5 | 2 trials based on vision and auditory (in vitro cues) | The virtual reality visor was placed immediately prior to the start of the cue presentation procedure and participants then proceeded through four virtual “rooms”. The first and last of these rooms had neutral cues (a TV displaying wildlife images) and the middle two had smoking cues. In one of the smoking cue rooms, participants navigated around a room containing a variety of objects commonly associated with smoking such as cigarette packs, ash trays, and burning cigarettes. The other smoking cue room contained people smoking, talking about smoking, and drinking. The sensations were primarily visual with some auditory input that included a voice-over providing information regarding the wildlife images displayed in the neutral rooms, and music and / or virtual people speaking in the cue rooms [80]. |
| 6 | 1 trial based on vision (in vivo cues) | On each of multiple Choice Behavior Under Cued Conditions (CBUCC) trials, participants are exposed to an in vivo cue (e.g., a lit cigarette, a cup of water). After rating craving in the presence of the cue, the participant spends real money ($0.01 to $0.25) to gain access to the cue; the more the participant spends, the greater the probability that the door will be unlocked and the cue can be sampled on that trial (probabilities range from 5 to 95%) [49]. |
| 7 | 1 trial based on behaviour, auditory and vision (in vivo/vitro cues) | The cue exposure therapy had three components: exposure to slides of smoking (visual) exposure to emotions and imagined situations that most reliably triggered an urge to smoke (emotional/imaginal), and exposure to a participant’s own cigarettes and pack (in vivo) [81]. |
| 8 | 1 trial based on behaviour and vision (in vivo/vitro cues) | Four conditions (phase 1): stress task and smoking cue, stress task and neutral cue, non-stressful task and smoking cue, or non-stressful task and neutral cue. Physiological and craving measures were collected and followed by a 10-minute break. All participants then went through the extinction protocol (phase 2) which entailed four rotations of: a five-minute video with smoking-related content (composed of similar but non-identical clips to those presented in the baseline visit), a five-minute presentation of smoking images (with each image presented for 3 seconds, see Supplementary Methods), and five minutes of manipulating smoking paraphernalia (e.g., lighter, cigarettes) [96]. |
| 9 | 1 trial based on behaviour (in vivo/vitro cues) | On imagery trials, scripts were presented over headphones. Participants had their eyes closed throughout the imagery procedure. The three cigarette imagery scripts contained explicit craving descriptors, and each included descriptions of watching people smoke to provide overlap with the primary stimulus content of the cigarette in vivo trials. The three neutral imagery scripts were devoid of any craving or smoking content. During the six in vivo trials, the participant opened his or her eyes when cued by a tone presented over the headphones. The participant then observed a same-gender experimenter, seated 10 ft (3 m) away, either lighting and smoking the participant's brand of cigarettes or pouring a glass of water and drinking from the glass. At the end of the cue-exposure period, the participant was signaled to close his or her eyes and think about what he or she had observed until hearing the word *stop*. The sequence of events for the in vivo trials paralleled the imagery trial sequence: 30 s of baseline, 50 s of cue exposure, 30 s of thinking about the cue presentation, and 30 s of relaxation [70]. |
